# The phosphoenolpyruvate carboxykinase (PEPCK) inhibitor, 3-mercaptopicolinic acid (3-MPA), induces myogenic differentiation in C2C12 cells

**DOI:** 10.1038/s41598-020-79324-9

**Published:** 2020-12-17

**Authors:** Madelaine C. Brearley, Zoe C. T. R. Daniel, Paul T. Loughna, Tim Parr, John M. Brameld

**Affiliations:** 1 School of Biosciences, University of Nottingham, Sutton Bonington Campus, Loughborough, Leicestershire, LE12 5RD UK; 2 School of Veterinary Medicine and Science, University of Nottingham, Sutton Bonington Campus, Loughborough, Leicestershire, LE12 5RD UK; 3 Present Address: Department of Medicine-Cardiology, University of California, Los Angeles, CA USA

**Keywords:** Differentiation, Metabolism

## Abstract

Phosphoenolpyruvate carboxykinase (PEPCK) is a gluconeogenic enzyme with a cytosolic (*Pck1*/PEPCK-C) and mitochondrial (*Pck2*/PEPCK-M) isoform. Here we investigate the effect of 3-mercaptopicolinic acid (3-MPA), a PEPCK inhibitor, on C2C12 muscle cells. We report that *Pck2* mRNA is 50–5000-fold higher than *Pck1* during C2C12 myogenesis, indicating *Pck2* is the predominant PEPCK isoform. C2C12 cell proliferation was inhibited in a dose-dependent manner following 48 h 3-MPA treatment (0.01–1 mM). C2C12 myogenic differentiation was significantly induced following 3-MPA treatment (0.25, 0.5, 1 mM) from day 0 of differentiation, demonstrated by increased creatine kinase activity, fusion index and myotube diameter; likewise, the myosin heavy chain (MyHC)-IIB isoform (encoded by *Myh4)* is an indicator of hypertrophy, and both porcine *MYH4*-promoter activity and endogenous *Myh4* mRNA were also significantly induced. High doses (0.5 and/or 1 mM) of 3-MPA reduced mRNA expression of *Pck2* and genes associated with serine biosynthesis (Phosphoglycerate dehydrogenase, *Phgdh*; phosphoserine aminotransferase-1, *Psat1*) following treatment from days 0 and 4. To conclude, as *Pck2*/PEPCK-M is the predominant isoform in C2C12 cells, we postulate that 3-MPA promoted myogenic differentiation through the inhibition of PEPCK-M. However, we were unable to confirm that 3-MPA inhibited PEPCK-M enzyme activity as 3-MPA interfered with the PEPCK enzyme assay, particularly at 0.5 and 1 mM.

## Introduction

We previously reported that β_2_-adrenergic receptor agonist (BA)-induced muscle hypertrophy in pigs was associated with a coordinated upregulation of a novel group of genes associated with an integrated stress response^1,2^, including mitochondrial phosphoenolpyruvate carboxykinase (*Pck2*) and genes involved in serine biosynthesis (phosphoglycerate dehydrogenase, *Phgdh*; phosphoserine aminotransferase-1, *Psat1*). We also reported a coordinated upregulation in mRNA expression of this same group of genes at day 2 of differentiation in C2C12 muscle cells, which coincided with the peak in myogenin expression^3^, suggesting that they might be important for myogenic differentiation.

Phosphoenolpyruvate carboxykinase (PEPCK) is a gluconeogenic enzyme that converts oxaloacetate to phosphoenolpyruvate (PEP), but is also involved in amino acid synthesis, glyceroneogenesis and cataplerosis^4^. There are two isoforms: the cytosolic isoform (PEPCK-C) encoded by *Pck1* and the mitochondrial isoform (PEPCK-M) encoded by *Pck2*. Previous studies looking into PEPCK function focussed on the role of PEPCK-C in hepatic gluconeogenesis and its involvement in type-2 diabetes, due to its tight hormonal regulation^4^. Although PEPCK-M was found to be constitutively expressed, it was classed as the ‘inferior’ isoform and its specific role was overlooked for many years, until recently when it was identified as an important regulator of cancer cell metabolism^5,6^. 3-Mercaptopicolinic Acid or 3-Mercaptopicolinate (3-MPA), a known inhibitor of gluconeogenesis, has been reported to inhibit both isoforms of PEPCK, but with increased potency for PEPCK-C *in vitro*^7–9^. The aim of this study was to compare the endogenous expression of *Pck1* and *Pck2* in proliferating and differentiating C2C12 cells and then determine the dose-dependent effects of the PEPCK inhibitor, 3-MPA, on proliferation, differentiation and gene expression of C2C12 cells.

## Results

### Treatment with 3-MPA inhibits proliferation of C2C12 cells

**Figure 1:**
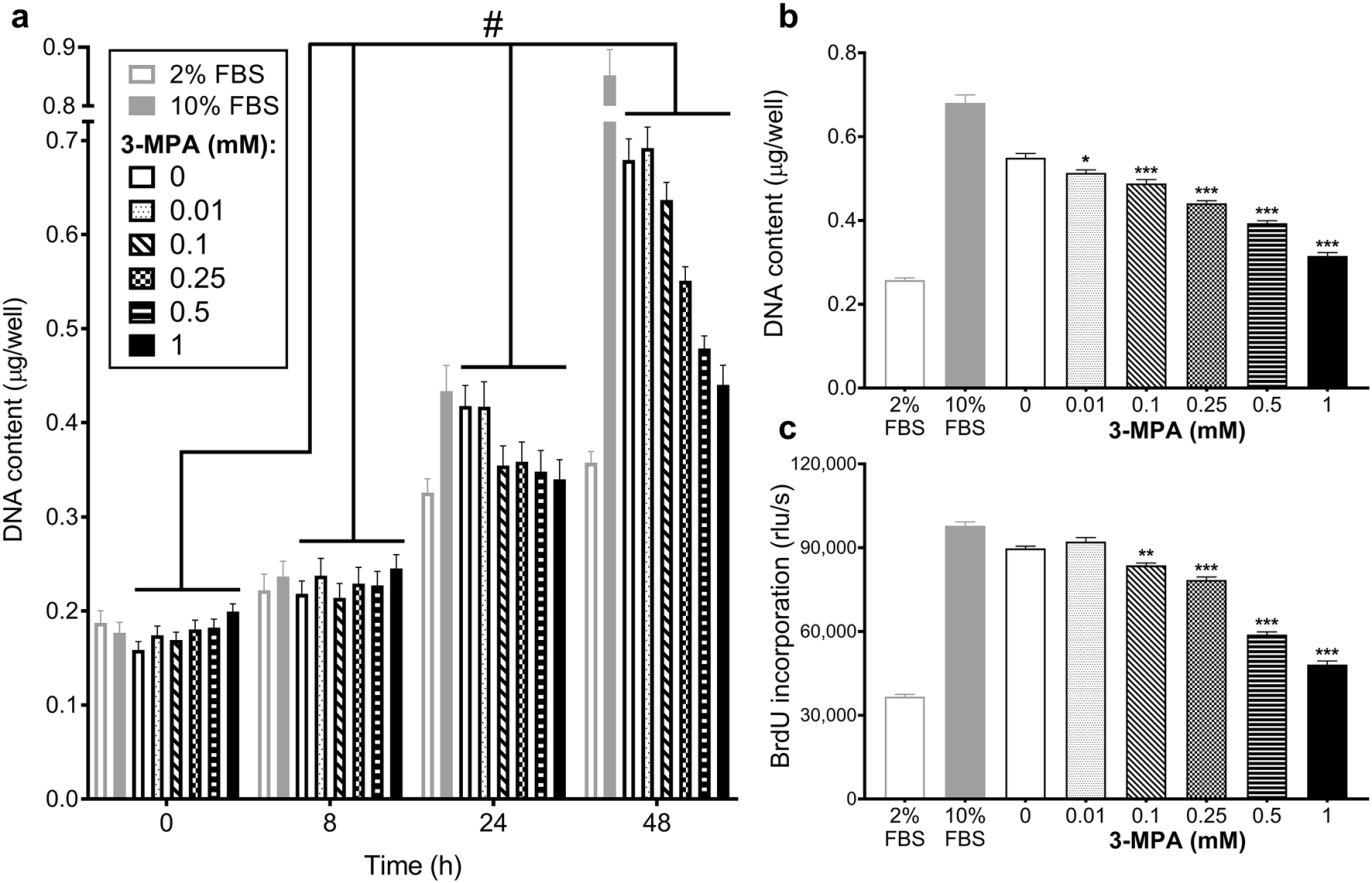
Treatment with 3-MPA reduced C2C12 cell number by inhibiting proliferation. Proliferating C2C12 cells were treated with a range of 3-MPA doses (0.01–1 mM) or equivalent volume of vehicle control (PBS; 0 mM 3-MPA) diluted in GM supplemented with 5% (v/v) FBS. (**a**) Cells were collected for total DNA quantification after 0, 8, 24 and 48 h of treatment. Data presented as Means (n = 16) ± SEM. Two-way ANOVA was performed: ^#^*P* < 0.001 (time × treatment interaction). Cells were also either (**b**) collected to quantify total DNA content after 48 h or (**c**) incubated with BrdU labelling solution for the final 3 h of the 48 h period to quantify BrdU incorporation. The data presented in (**a**) and (**b**) were obtained in separate experiments. Data presented as Means (n = 8) ± SEM. One-way ANOVA was performed, followed by a Dunnett’s multiple comparison to vehicle control (0 mM 3-MPA): *P < 0.05, **P < 0.01 and ***P < 0.001. 2% and 10% (v/v) FBS were internal controls and therefore excluded from the statistical analyses.

C2C12 cells were treated with a range of 3-MPA doses (0–1 mM) and total DNA content quantified as a marker of total cell number. There was a significant time × treatment interaction (*P* < 0.001), with 3-MPA inducing a dose-dependent (0.1–1 mM) reduction in total DNA content compared to the vehicle control (PBS; 0 mM 3-MPA) but only after 24 and 48 h treatment (Fig. 1a). In the presence of 1 mM 3-MPA, total DNA content increased with time and was higher than cells treated with 2% (v/v) FBS, particularly after 48 h. This suggests that there were no or limited toxic effects within the range of 3-MPA doses used, although cell viability or toxicity were not determined. The reduction in cell number after 48 h of treatment (Fig. 1b) is consistent with the significant reduction in BrdU incorporation (Fig. 1c), particularly with the higher doses of 3-MPA (0.1–1.0 mM). This suggests that treatment with 3-MPA reduces total cell number in a dose-dependent manner, probably as a result of inhibiting cell proliferation.

### Treatment with 3-MPA induces myogenic differentiation of C2C12 cells

**Figure 2:**
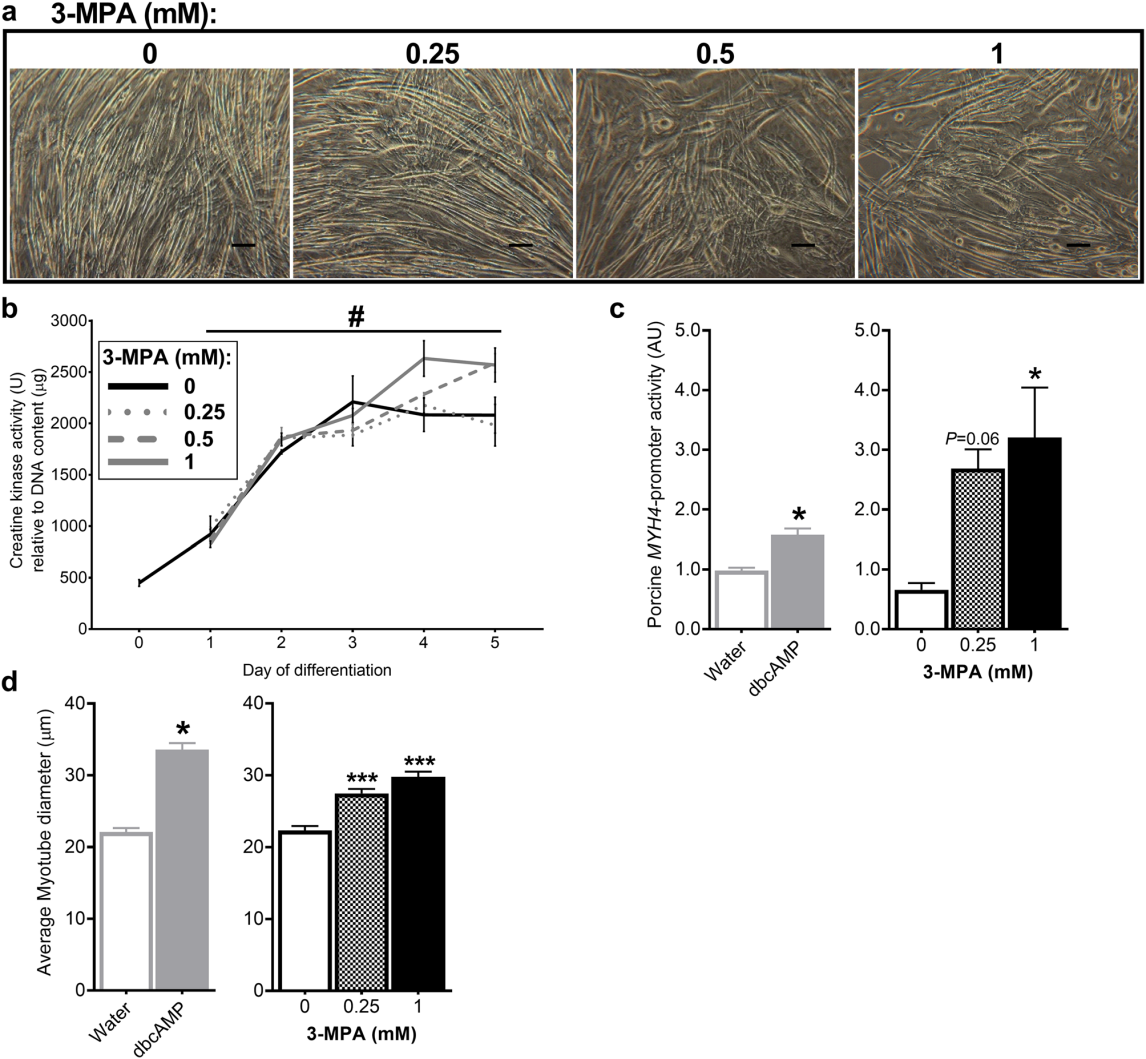
Treatment with 3-MPA induces myogenic differentiation. C2C12 cells were treated with 0, 0.25, 0.5 or 1 mM 3-MPA from day 0 of differentiation for five days, with DM refreshed every 48 h. Samples were collected every 24 h for six days and a Creatine Kinase (CK) activity assay performed. (**a**) Representative bright-field images captured at day 5 of treatment with 0, 0.25, 0.5 or 1 mM 3-MPA. Photographs were captured at ×6.3 magnification. Scale bar: 100 µm. Data presented as Means (n = 3, except day 0 n = 6) ± SEM for (**b**) CK activity normalised to DNA content. ^#^*P* < 0.05 (time × treatment interaction). C2C12 cells were transfected with *MYH4*-ZsGreen expression plasmid on day − 1, then treated with 0, 0.25 or 1 mM 3-MPA from day 0 until day 4 of differentiation. Measurements were taken for (**c**) porcine *MYH4-*promoter activity and (**d**) average myotube diameter. Data presented as Means ± SEM, from three wells for *MYH4-*promoter activity (n = 3) and five separate fields of view per well for myotube diameter (n = 15). (**c**) Student’s t-test was used for dbcAMP treatment and one-way ANOVA for 3-MPA doses and (**d**) one-way ANOVA (blocking for ‘well’) were performed for each treatment group and appropriate vehicle controls, followed by a post-hoc Dunnett’s multiple comparison test for 3-MPA doses compared to the vehicle control (PBS; 0 mM 3-MPA). *P < 0.05, ***P < 0.001.

Treatment of C2C12 cells with increasing doses of 3-MPA (0.25, 0.5 and 1 mM) from day 0 of differentiation resulted in larger myotubes being observed at day 5 (Fig. 2a). With increasing doses of 3-MPA, cells became sparser but resulted in more round bulging cells and much larger myotubes. These observations are comparable to those reported previously by our group in C2C12 cells treated with dbcAMP from day 0 of differentiation^3^. Creatine kinase (CK) activity is an established indicator of myogenic differentiation, therefore we next quantified CK activity every 24 h following treatment with 3-MPA from day 0 until day 5. There was a significant time × treatment interaction for CK activity normalised for DNA (*P* = 0.014; Fig. 2b), due to cells treated with 0.5 or 1 mM 3-MPA having increased CK activity at days 4 and 5, indicating that these higher doses of 3-MPA induced greater myogenic differentiation compared to the control.

Myosin Heavy Chain (MyHC)-IIB isoform expression is often associated with hypertrophic growth. We recently reported the response of the porcine *MYH4* promoter to various known anabolic and catabolic agents using a fluorescence-based promoter-reporter system^10^, here we utilise the same system to determine the effects of 3-MPA treatment on both porcine *MYH4*-promoter activity and myotube diameters simultaneously. dbcAMP treatment was used as a positive control and, as expected, dbcAMP treatment significantly increased both *MYH4*-promoter activity (*P* = 0.007) and myotube diameters (*P* = 0.009). Similarly, 3-MPA treatment (at 0.25 and 1 mM) also induced both porcine *MYH4*-promoter activity (*P* = 0.03; Fig. 2c) and myotube diameter (*P* < 0.001; Fig. 2d).

### Treatment with 3-MPA increases fusion index in C2C12 cells

**Figure 3:**
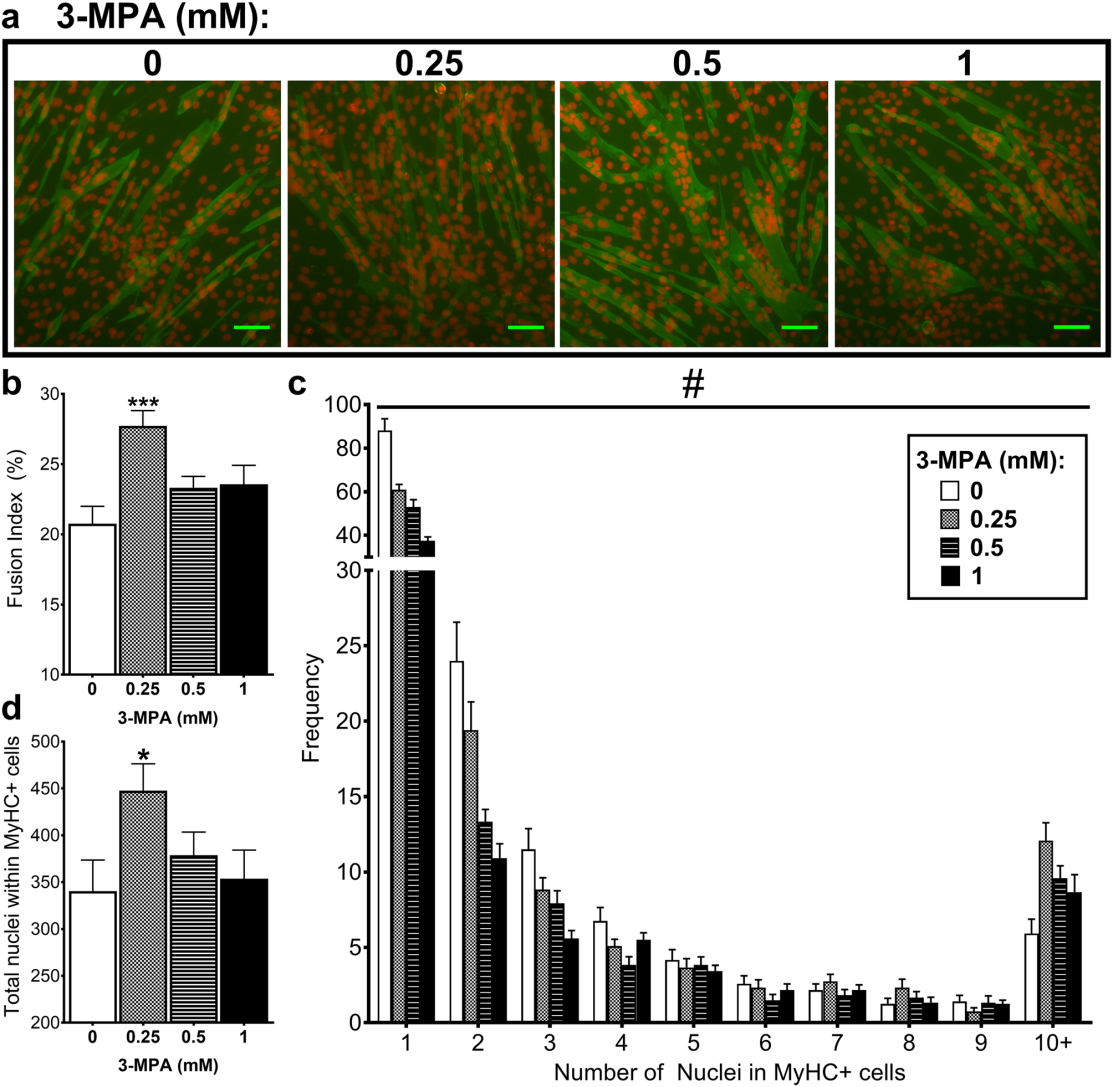
Treatment with 3-MPA increases fusion index. C2C12 cells were treated with 0, 0.25, 0.5 or 1 mM 3-MPA from day 0 of differentiation, then after 72 h treatment immunofluorescence was performed using MyHC antibody (green) and PI (red). (**a**) Photographs captured at 10X magnification. Scale bar: 100 µm. ImageJ software was used to quantify (**b**) Fusion Index, (**c**) the frequency of nuclei found within MyHC+ cells and (**d**) total nuclei number within MyHC  cells. Data presented as Means (n = 12, from four wells per treatment and three fields of view per well) ± SEM. One- or two-way ANOVAs (blocking for ‘well’) were performed as appropriate, followed by Dunnett’s comparison test to the vehicle control (PBS; 0 mM 3-MPA). ^#^*P* < 0.001 (treatment × nuclei number interaction) *P < 0.05, ***P < 0.001.

Fusion index is the percentage of nuclei per field of view found inside MyHC positive (MyHC+) cells and is another indicator of myogenic differentiation (Fig. 3a). Average fusion index was significantly increased following 0.25 mM 3-MPA treatment compared to control (*P* < 0.001; Fig. 3b). There was a significant treatment × number of nuclei interaction (*P* < 0.001; Fig. 3c), whereby the frequency of MyHC+ cells containing one, two or three nuclei demonstrated a dose-dependent reduction with increasing doses of 3-MPA compared to the control; however, the frequency of MyHC+ cells containing 10+ nuclei was increased with 3-MPA treatment, particularly at 0.25 mM. MyHC+ cells treated with 3-MPA were observed to contain 40 or more nuclei, whereas control cells showed a maximum of ~ 20 nuclei. Similarly, the total number of nuclei within MyHC+ cells was significantly affected by 3-MPA treatment (*P* = 0.02; Fig. 3d) where 0.25 mM, but not 0.5 or 1 mM, resulted in a significant increase in the total number of nuclei within MyHC+ cells.

### Treatment with 3-MPA alters mRNA expression of genes associated with myogenesis

**Figure 4:**
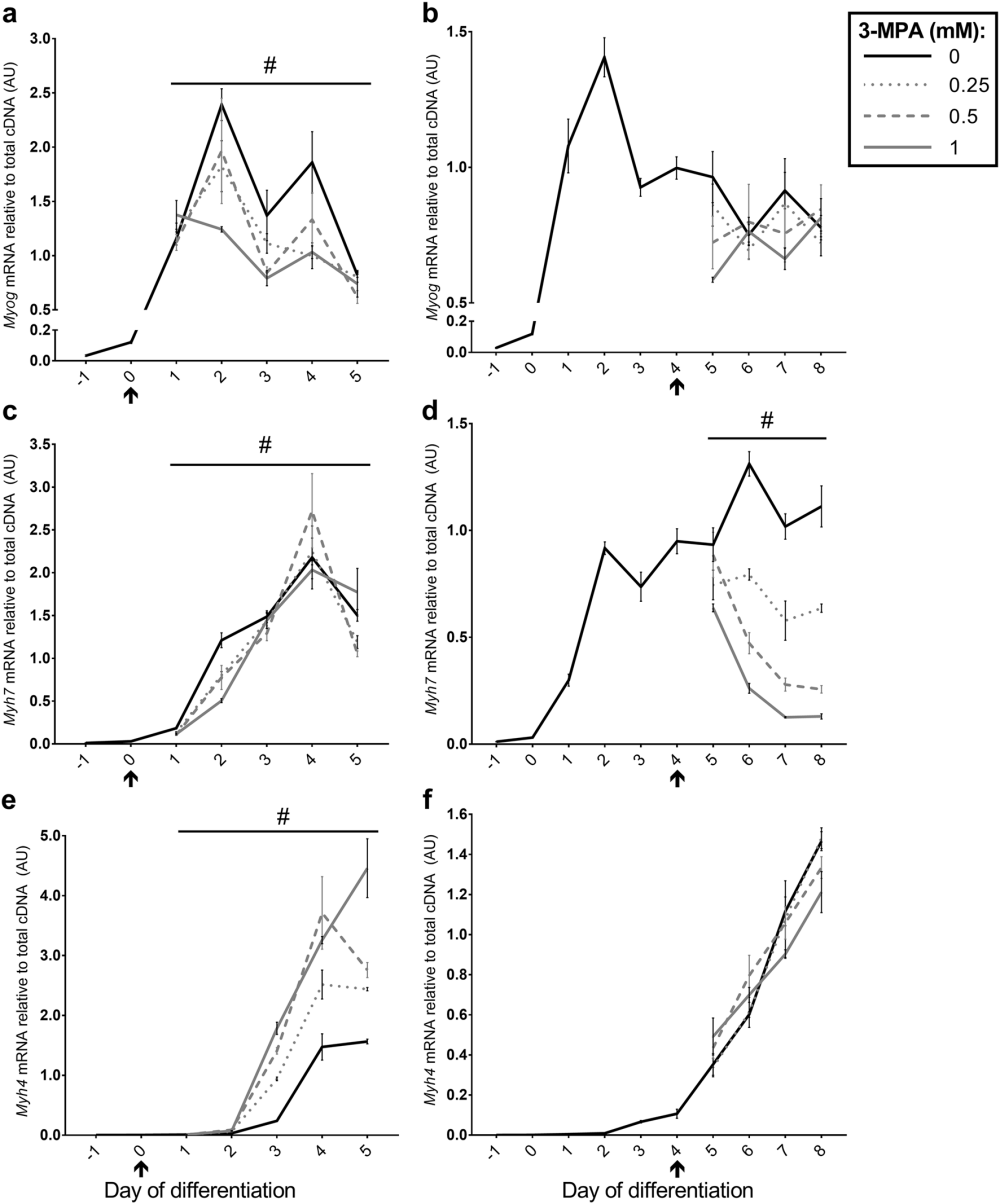
Effect of 3-MPA treatment on genes associated with myogenesis. Relative expression of (**a**, **b**) *Myog* (Myogenin), (**c**, **d**) *Myh7* (encodes Myosin Heavy Chain (MyHC)-I), and (**e**, **f**) *Myh4* (encodes MyHC-IIB) mRNA following treatment of C2C12 cells with 0, 0.25, 0.5 or 1 mM 3-MPA from day 0 (**a**, **c**, **e**) or day 4 (**b**, **d**, **f**) of differentiation. Data presented as Means (n = 4–6) ± SEM. ^#^*P* < 0.05 (time × treatment interaction). Start of treatment indicated by ↑. See Supplementary Tables 1 and 2 for Dunnett’s Multiple Comparison tests.

There was a significant time × treatment interaction (*P* = 0.02) for myogenin (encoded by *Myog*) mRNA expression following treatment with 3-MPA (0.25–1 mM) from day 0 of differentiation, but not from day 4 (*P* = 0.177). As observed previously^3^, myogenin mRNA peaked at day 2 of differentiation then decreased with differentiation (Fig. 4a). Treatment with 1 mM 3-MPA from day 0 resulted in lower myogenin mRNA compared to the control at days 2 and 4; however, there was no effect of treatment (*P* = 0.118; Fig. 4b) or time (*P* = 0.859) following 3-MPA treatment from day 4. See Supplementary Table 1 for a summary of Dunnett’s Multiple Comparison tests.

There were significant time × treatment interactions for *Myh7* (encodes slow oxidative MyHC-I isoform) mRNA following 3-MPA treatment from both day 0 (*P* = 0.008, Fig. 4c) and day 4 (*P* < 0.001, Fig. 4d). As observed previously^3^, *Myh7* mRNA was expressed from day 1, whereas 3-MPA treatment from day 0 generally reduced *Myh7* mRNA after 48 h treatment (day 2) at all doses, particularly 1 mM, but had inconsistent effects at other time points (Fig. 4c). In contrast, treatment with 3-MPA from day 4 of differentiation resulted in a clear dose-dependent reduction in *Myh7* mRNA between days 6 and 8 (Fig. 4d). See Supplementary Tables 1 and 2 respectively for summaries of Dunnett’s Multiple Comparison tests following treatment from day 0 and day 4. There was also a significant time × treatment interaction for *Myh4* (encodes fast glycolytic MyHC-IIB isoform) mRNA following 3-MPA treatment from day 0 (*P* < 0.001, Fig. 4e), however only a trend was observed following treatment from day 4 (*P* = 0.097, Fig. 4f). Treatment with 3-MPA from day 0 resulted in a dose-dependent increase in *Myh4* mRNA from day 3 onwards (Fig. 4e); whereas there was a significant increase in *Myh4* mRNA expression with time (*P* < 0.001; Fig. 4f), but no effect of 3-MPA treatment from day 4 (*P* = 0.548).

### Endogenous mRNA expression of PEPCK isoforms during differentiation of C2C12 cells

The level of cytosolic phosphoenolpyruvate carboxykinase (PEPCK-C encoded by *Pck1*) isoform mRNA was very lowly expressed in C2C12 cells and consequently an accurate standard curve could not be generated for this gene. As a result we compared crossing point (Cp) values for the two PEPCK isoforms to approximate changes in transcript levels during myogenic differentiation. In proliferating myoblasts (days − 1 and 0), the mitochondrial PEPCK isoform (PEPCK-M encoded by *Pck2*) was over 2500-fold (11.5 Cp values; Table 1) higher than *Pck1* and on average over 200-fold (8 Cp values, Table 1) higher in differentiated myotubes (days 4–8); therefore, this indicates that *Pck2* is the predominant PEPCK isoform in C2C12 cells.

**Table 1:** Average Crossing Point (Cp) values for murine *Pck1* and *Pck2* mRNA expression in C2C12 cells during myogenic differentiation.

Day	− 1	0	1	2	3	4	5	6	7	8
*Pck1*	37.5	37.2	36.2	35.8	35.9	35.4	35.6	36.7	36.8	34.0
*Pck2*	25.9	25.7	27.1	26.6	27.0	27.6	27.4	27.8	27.4	28.1

### Treatment with 3-MPA alters mRNA expression of genes associated with gluconeogenesis and serine biosynthesis

**Figure 5:**
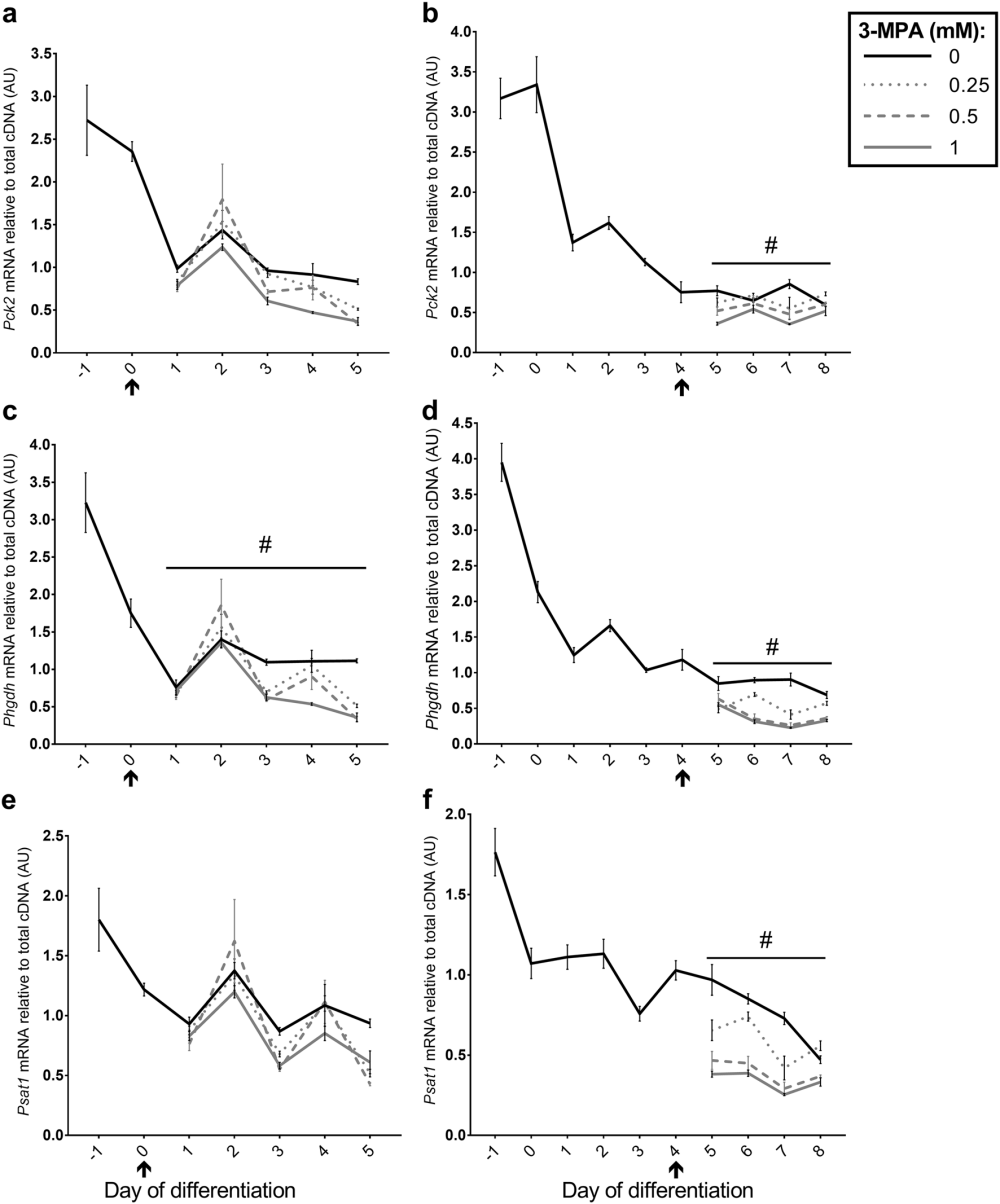
Effect of 3-MPA treatment on genes associated with gluconeogenesis and serine biosynthesis. Relative expression for (**a**, **b**) *Pck2* (encodes mitochondrial PEPCK isoform), (**c**, **d**) phosphoglycerate dehydrogenase (*Phgdh)* and (**e**, **f**) phosphoserine aminotransferase-1 (*Psat1)* mRNA following treatment of C2C12 cells with 0, 0.25, 0.5 or 1 mM 3-MPA from day 0 (**a**, **c**, **e**) or day 4 (**b**, **d**, **f**) of differentiation. Data presented as Means (n = 4–6) ± SEM. ^#^*P* < 0.05 (time × treatment interaction). Start of treatment indicated by ↑. See Supplementary Tables 1 and 2 for Dunnett’s Multiple Comparison tests.

There was no time × treatment interaction for *Pck2* mRNA expression following treatment from day 0 (*P* = 0.119), however there were significant effects of time and 3-MPA treatment separately (both *P* < 0.001; Fig. 5a). Relative mRNA expression of *Pck2* was higher in proliferating myoblasts than differentiated myotubes and a peak at day 2 of differentiation was observed that coincided with the peak in myogenin mRNA, as reported previously^3^. There was a significant time × treatment interaction on *Pck2* mRNA expression following 3-MPA treatment from day 4 (*P* = 0.006; Fig. 5b), with 0.5 and 1 mM 3-MPA reducing *Pck2* mRNA at days 5 and 7, whereas treatment with 0.25 mM 3-MPA only reduced *Pck2* mRNA at day 7.

There was a significant time × treatment interaction for phosphoglycerate dehydrogenase (*Phgdh*) mRNA expression following 3-MPA treatment from day 0 and day 4 (both *P* < 0.001; Fig. 5c,d). Relative *Phgdh* mRNA was higher in proliferating myoblasts at day − 1 than differentiated myotubes and indicated a similar day 2 peak that coincided with myogenin and *Pck2* mRNA, as observed previously^3^. Treatment with 3-MPA (0.25, 0.5 and 1 mM) from day 0 of differentiation generally reduced *Phgdh* mRNA between days 3 and 5 (Fig. 5c), whereas 3-MPA treatment from day 4 reduced *Phgdh* mRNA at all time-points (between days 5 and 8; Fig. 5d).

There was no time × treatment interaction for phosphoserine aminotransferase-1 (*Psat1*) mRNA expression following 3-MPA treatment from day 0 (*P* = 0.109), but there were significant effects of time (*P* < 0.001) and 3-MPA (*P* = 0.018) separately. Relative mRNA expression of *Psat1* was higher in proliferating myoblasts at day − 1 than differentiated myotubes (Fig. 5e,f) and treatment with 3-MPA (0.25, 0.5 and 1 mM) generally reduced *Psat1* at days 3 and 5 (Fig. 5e). There was a significant time × treatment interaction for *Psat1* mRNA following 3-MPA treatment from day 4 (*P* < 0.001), with 3-MPA treatment (0.25, 0.5 and 1 mM) reducing *Psat1* mRNA between days 5 and 7 (Fig. 5f).

## Discussion

For the first time we report the effects of the gluconeogenic inhibitor, 3-mercaptopicolinic acid (3-MPA), on proliferation and myogenic differentiation of C2C12 cells. 3-MPA treatment (0.01–1 mM) inhibited cell proliferation (total DNA and BrdU incorporation) in a dose-dependent manner and induced myogenic differentiation (CK assay) at 0.5 and 1 mM. Interestingly, 3-MPA resulted in similar effects to those observed previously with dbcAMP treatment of C2C12 cells from day 0^3^; including significant increases in myotube diameters, porcine *MYH4*-promoter activity and endogenous *Myh4* mRNA expression. These results were contrary to our original hypothesis that mitochondrial phosphoenolpyruvate carboxykinase (*Pck2*/PEPCK-M) was important for myogenesis and therefore inhibition would result in myotube atrophy. However they do agree with our recent finding that *Pck2* overexpression in mouse skeletal muscle, via intramuscular injection of an AAV construct, results in a decrease in muscle weights^11^.

We also make the novel observation that mitochondrial *Pck2* is the predominant PEPCK isoform in C2C12 cells, with cytosolic *Pck1* mRNA being significantly lower (50–5000-fold) than *Pck2* mRNA throughout C2C12 myogenic differentiation. Therefore, we suggest that the phenotypic responses observed in this study were probably a result of PEPCK-M inhibition, although this still requires confirmation. The effect of 3-MPA on mRNA transcript abundance was different following treatment of C2C12 cells at the onset of differentiation (from day 0) compared to treatment of differentiated myotubes (from day 4), with effects mainly observed with the higher doses of 3-MPA (0.5 and 1 mM). We previously reported coordinated increases in *Pck2* mRNA and the genes involved in serine biosynthesis (Phosphoglycerate dehydrogenase, *Phgdh*; Phosphoserine aminotransferase-1, *Psat1*) following BA-induced hypertrophy in porcine skeletal muscle^1^, as well as during C2C12 cell myogenic differentiation^3^. The data presented here supports the synchronous nature and possible coordinated regulation of *Pck2* mRNA and the serine biosynthesis genes (Phgdh and Psat1), since treatment with 3-MPA from day 0 or day 4 resulted in significant reductions in *Pck2*, *Phgdh* and *Psat1* mRNA expression, albeit at slightly different timepoints (Fig. 5).

Treatment with 3-MPA from day 0 or day 4 of differentiation either reduced or had no effect on myogenin mRNA respectively. However, 3-MPA significantly increased *Myh4* mRNA, the Myosin Heavy Chain (MyHC)-IIB isoform often associated with hypertrophy, particularly with treatment from day 0. This is similar to the previously observed effects of dbcAMP^3^, however there were no effects when C2C12 myotubes were treated with 3-MPA from day 4. In contrast, 3-MPA treatment from day 0 had little effect on *Myh7* (encodes slow oxidative MyHC-I isoform) mRNA, whereas treatment with increasing doses of 3-MPA from day 4 induced a dose-dependent reduction in *Myh7* mRNA.

Computational analysis of the structure for rat cytosolic PEPCK (i.e. *Pck1*) indicate that 3-MPA binds to both the PEP/OAA active site and an allosteric site that helps stabilise the enzyme^12^. It is unclear whether the same mechanism applies to mitochondrial PEPCK (PEPCK-M/ *Pck2*), but 3-MPA treatment of lung cancer cell lines induced similar responses to that of PEPCK-M gene specific knock-down^6^. Since we show that PEPCK-M is the predominant PEPCK isoform in C2C12 cells, we assume that the responses observed are mainly due to inhibition of PEPCK-M. It has previously been reported that 3-MPA inhibits PEPCK activity^13^, but the highest dose studied was 0.1 mM. We found that 3-MPA appeared to interfere with the PEPCK enzymatic activity assay used previously by our group^11^, particularly at the higher doses > 0.25 mM (see Supplementary Tables 3, 4 and 5). Therefore, it is unclear whether or not the effects of 3-MPA on C2C12 cell proliferation, differentiation, hypertrophy and gene expression are due to inhibition of PEPCK-M activity. Although 3-MPA has been reported as a highly selective PEPCK inhibitor at 0.3 mM in *Trypanosoma cruzi*, with minimal or no effects on other enzymes^14^, it has also been shown that millimolar concentrations of 3-MPA inhibit Glucose-6-phosphate translocase in isolated rat livers^15^. Therefore, it is possible that the responses to the higher doses (0.5 and 1 mM) of 3-MPA observed in C2C12 cells may be due to effects on other enzymes.

## Conclusions

Contrary to initial expectations, treatment of C2C12 cells with 3-MPA induced myogenic differentiation, including increases in CK activity, fusion index and myotube diameters, as well as increasing porcine *MYH4*-promoter activity and endogenous *Myh4* mRNA expression. This induction of differentiation was associated with a concomitant decrease in cell proliferation, which is as expected, given that C2C12 cells need to exit the cell cycle and therefore stop proliferating before they can terminally differentiate into myotubes. These responses are similar to the effects of dbcAMP on C2C12 cells we previously reported^3^ and do agree with our recent observation that *Pck2* overexpression in mouse muscle results in reduced muscle weights^11^. However, as 3-MPA interfered with the PEPCK activity assay, it is unclear whether these novel observations, particularly at higher doses (0.5 and 1 mM), are due to PEPCK-M inhibition or a result of off-target effects, but the pro-myogenic effect of 3-MPA was accompanied by inhibition of genes involved in gluconeogenesis and serine synthesis. It would be useful to determine if 3-MPA inhibits PEPCK-M activity at these higher doses, as well as any effects on other enzymes.

## Methods

### 3-MPA treatment

3-MPA (SC-206655, Santa Cruz Biotechnology) was dissolved in phosphate buffered saline (PBS), filtered through a 20 µm filter and stock solution aliquots (20 mM) stored at − 20 °C. 3-MPA was diluted firstly in PBS followed by a 1:10 dilution into culture media, providing a concentration range of 0.01 to 1 mM, based on previously reported in vitro studies^8,9,16^. C2C12 cell stocks were originally purchased from the European Collection of Authenticated Cell Cultures (ECACC; 91031101) and checked regularly for mycoplasma. They were initially cultured in Growth Medium (GM; high (25 mM) glucose Dulbecco’s modified eagle’s medium (DMEM; Sigma-Aldrich, Poole, UK) supplemented with 10% (v/v) foetal bovine serum (FBS) and 1% (v/v) penicillin/streptomycin (P/S)). Once the cells reached ~ 80% confluence (day 0), they were switched to Differentiation Medium (DM; high glucose (25 mM) DMEM supplemented with 2% (v/v) horse serum (HS) and 1% (v/v) P/S). Media was refreshed every 48 h. All treatments were made up fresh on the day of treatment and equivalent volume of PBS (0 mM 3-MPA) was used as the vehicle control, such that the dilution of the media was constant for all treatments.

### Total DNA content and BrdU incorporation assay

C2C12 cells were seeded at 2500 cells per well onto black-walled, clear-bottomed 96-well plates and left to settle overnight. Cells were treated at ~ 20% confluence with a range of 3-MPA doses (0–1 mM) added to GM supplemented with 5% (v/v) FBS for up to 48 h, then assays were performed directly onto 96-well plates. DNA assays were performed as described previously^17,18^ and BrdU incorporation assay was carried out according to the manufacturers guide for the Cell Proliferation (chemiluminescent) ELISA kit (Roche Diagnostics, Mannheim, Germany). Cells were treated with GM supplemented with either 2% or 10% (v/v) FBS to act as internal controls and determine the effective range for the two assays.

### Creatine kinase activity assay

C2C12 cells were seeded at 150,000 cells per well onto 6-well plates. At ~ 80% confluence (day 0 of differentiation), cells were switched to DM supplemented with a range of 3-MPA doses (0–1 mM). DM was then refreshed every 48 h and wells harvested every 24 h from day 0 for six days to determine creatine kinase (CK) activity. CK activity assay was performed as described previously^17,18^.

### Fusion index assay

C2C12 cells were seeded at 60,000 cells per well of a 12-well plate and subsequently switched to DM supplemented with a range of 3-MPA doses (0–1 mM) at ~ 80% confluence (day 0 of differentiation). After 72 h treatment, C2C12 cells were fixed using ice cold 100% (v/v) methanol, washed in PBS, then blocked for 1 h in 1% (v/v) Triton-X (Sigma-Aldrich) and 10% HS. Cells were incubated at 4 °C overnight with Myosin antibody (Developmental Studies Hybridoma Bank, Iowa, USA) diluted 1:1000 in 0.5% (v/v) Triton-X and 10% (v/v) HS. Cells were washed in PBS, then incubated at room temperature for 2 h with 1:1000 diluted Alexa Fluor-488 conjugated secondary antibody (Thermo Fisher, Illinois, USA) to detect all Myosin Heavy Chain (MyHC) isoforms (green), referred to as MyHC+ cells. Propidium Iodide solution (PI; Sigma-Aldrich, Missouri, USA) was diluted 1:3000 and incubated with cells for 15 min to stain cell nuclei red. Photographs were captured using a Leica fluorescent microscope for three randomly selected fields of view per well and four wells per treatment (n = 12). Frequency of nuclei and total number of nuclei found within MyHC+ cells per field of view were determined using ImageJ software. Fusion Index (%) was quantified as follows:

$$Fusion\; Index \left( \% \right) = \frac{{\left( {Number \;of \;nuclei\; in\; MyHC^{ + } \;cells} \right)}}{Total\; nuclei \;per \;field \;of\; view} \times 100$$

### Porcine *MYH4*-promoter activity and myotube diameter

C2C12 cells were seeded at 25,000 cells per well of a 24-well plate and co-transfected with porcine *MYH4*-ZsGreen and CMV-DsRed expression plasmids between 50 and 60% confluence as previously described^10^. At ~ 80% confluence, cells were switched to DM (day 0 of differentiation) supplemented with 0, 0.25 or 1 mM 3-MPA. Media was refreshed every 48 h. Measurements of porcine *MYH4*-promoter activity and myotube diameters were quantified as described previously^10^.

### Real-time quantitative PCR (QPCR) analyses

C2C12 cells were seeded at 150,000 cells per well of a 6-well plate and switched to DM at ~ 80% confluence (day 0 of differentiation). Cells were then either treated with a range of 3-MPA doses (0–1 mM) from day 0 for five days or from day 4 for four days. DM was refreshed every 48 h. Wells were harvested into 200 µl RNase-free PBS every 24 h from day − 1 for the duration of each time-course. Total RNA was extracted using the High Pure RNA Isolation kit (Roche Diagnostics, Mannheim, Germany), then RNA was reverse transcribed into cDNA using random hexamer primers included in the RevertAid RT First Strand cDNA Synthesis kit (Thermo Scientific, Vilnius, Lithuania). Relative mRNA expression for each gene was determined by real-time quantitative PCR (QPCR) and normalised using OliGreen to quantify total cDNA, as described previously^17^. QPCR primers used in this study are reported elsewhere^3^.

### Statistical analyses

One- or two-way analysis of variance (ANOVA) was performed in GraphPad Prism (7.03) statistical software for data relating to the following assays: BrdU incorporation, porcine *MYH4* promoter activity, myotube diameter and QPCR. When appropriate (ANOVA *P* < 0.05), a post-hoc Bonferroni or Dunnett’s multiple comparison test was used to compare the 3-MPA doses and the vehicle control (PBS; 0 mM 3-MPA). For myotube diameters, one-way ANOVA was performed, blocking for ‘well’, to account for the different fields of view. A student’s t-test was performed to show the expected effects of dibutyryl cyclic-AMP (dbcAMP) treatment on porcine *MYH4*-promoter activity. Statistical analyses performed in GenStat (17th edition) include: two-way ANOVA (time × treatment) for CK activity and CK activity normalised to DNA content (blocking for ‘plate’); two-way ANOVA (time × treatment) for multiple time-point DNA assays, (blocking for experiment when data was pooled from two separate experiments); one-way ANOVA for fusion index, and two-way ANOVA for frequency of nuclei number in MyHC+ cells (blocking for ‘well’). Data are presented as Means ± standard error of the mean (SEM).

## Supplementary Information

Supplementary Information.

## Data Availability

The datasets generated and/or analysed during the current study are available from the corresponding author on reasonable request.
