# Supplementary material for: The phosphoenolpyruvate carboxykinase (PEPCK) inhibitor, 3-mercaptopicolinic acid (3-MPA), induces myogenic differentiation in C2C12 cells
Source: Sci Rep. 2020 Dec 17;10:22177. doi: 10.1038/s41598-020-79324-9 (PMC7747743; doi:10.1038/s41598-020-79324-9)
Supplement: Supplementary file 1 — Supplementary Information. [file 41598_2020_79324_MOESM1_ESM.docx]

**The** **phosphoenolpyruvate carboxykinase (PEPCK) inhibitor, 3-Mercaptopicolinic acid (3-MPA), induces myogenic differentiation in C2C12 cells**

Madelaine C. Brearley^a#^, Zoe C.T.R. Daniel^a^, Paul T. Loughna^b^, Tim Parr^a^ and John M. Brameld^a^*

^a^Schools of Biosciences & ^b^Veterinary Medicine & Science, University of Nottingham, Sutton Bonington Campus, Loughborough, Leicestershire, LE12 5RD, UK

Current address: ^#^Department of Medicine-Cardiology, University of California, Los Angeles, California, United States of America

*Corresponding author

Email: [John.Brameld@nottingham.ac.uk](mailto:John.Brameld@nottingham.ac.uk)

**Supplementary Figure 1. Microscope images of C2C12 cells cultured in different concentrations (2, 10 or 5% (v/v)) of Fetal Bovine Serum (FBS) with or without 3-MPA treatment (0, 0.1, 0.25, 0.5 or 1mM) from time 0.**

Representative bright-field images show proliferating C2C12 cells 8, 24 and 48 hours after treatment with a range of 3-MPA doses (0-1mM). Photographs were captured at 6.3X magnification. Scale bar: 100µm.

**
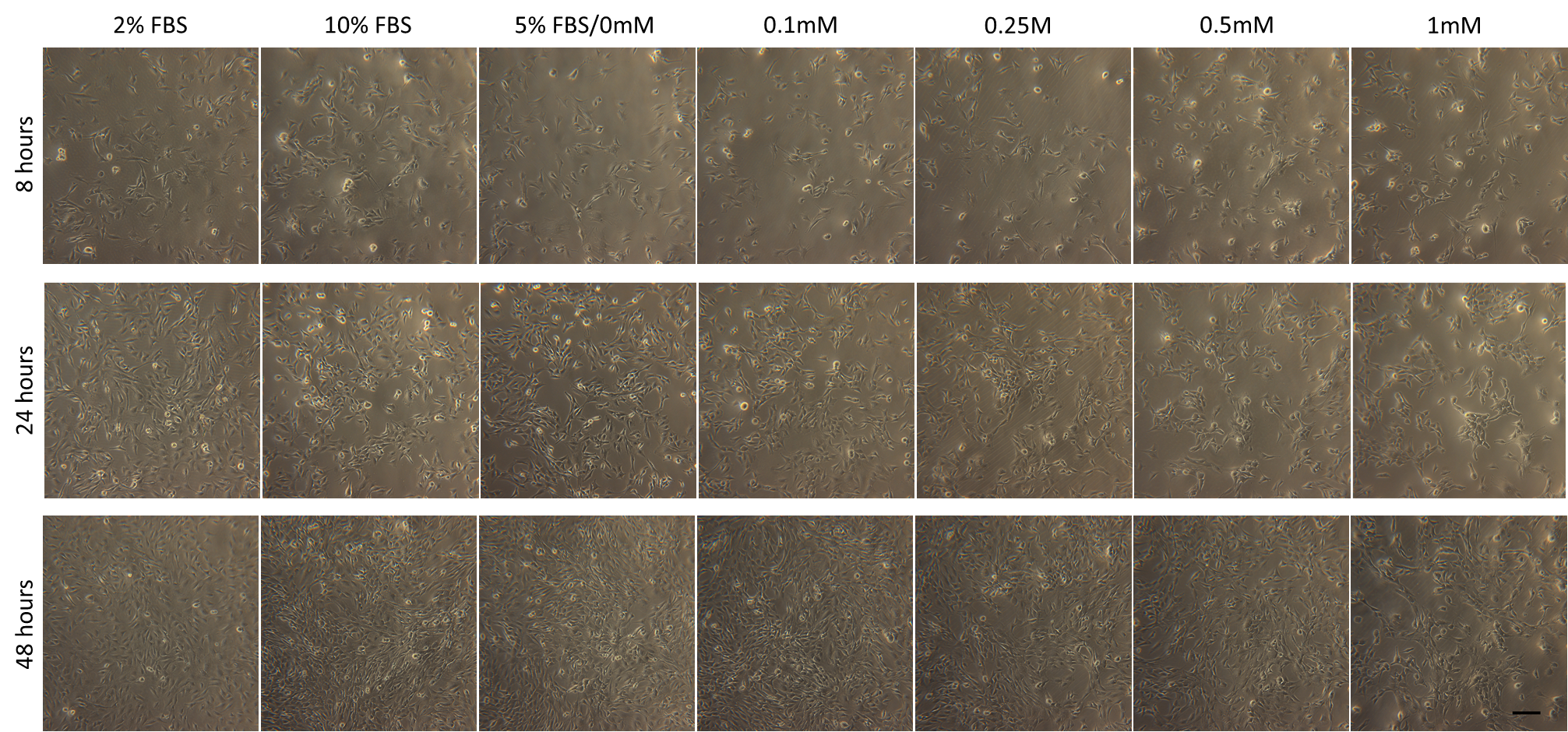
**

**Supplementary Table 1. Summary of post-hoc test analysis on Creatine Kinase (CK) normalised for DNA following 3-MPA treatment from day 0 of differentiation.**

|  | | **CK/DNA** |
| --- | --- | --- |
| **Time x Treatment interaction** | | *P*=0.014 |
| **Time** | **Treatment** |  |
| DAY 1 | Control | 920.9 |
| DAY 1 | 0.25 mM | 984.0 ns |
| DAY 1 | 0.5 mM | 874.1 ns |
| DAY 1 | 1 mM | 825.0 ns |
| DAY 2 | Control | 1723 |
| DAY 2 | 0.25 mM | 1856 ns |
| DAY 2 | 0.5 mM | 1870 ns |
| DAY 2 | 1 mM | 1845 ns |
| DAY 3 | Control | 2209 |
| DAY 3 | 0.25 mM | 2177 ns |
| DAY 3 | 0.5 mM | 2285 ns |
| DAY 3 | 1 mM | 2632 ns |
| DAY 4 | Control | 2083 |
| DAY 4 | 0.25 mM | 2177 ns |
| DAY 4 | 0.5 mM | 2285 ns |
| DAY 4 | 1 mM | **2632** ****** |
| DAY 5 | Control | 2080 |
| DAY 5 | 0.25 mM | 1983 ns |
| DAY 5 | 0.5 mM | **2589 *** |
| DAY 5 | 1 mM | **2569 *** |

Dunnett’s multiple comparison tests were performed on CK activity normalised to DNA following treatment with 0 (Control; PBS), 0.25, 0.5 or 1mM 3-MPA from day 0 of differentiation, demonstrating a time x treatment interaction (P<0.1). Data are means for n=3. Significant difference compared to control at each time-point is indicated: *P<0.05, **P<0.01.

**Supplementary Table 2. Summary of post-hoc test analysis on mRNA expression of genes following 3-MPA treatment from day 0 of differentiation.**

|  | | ***Myog*** | ***Myh7*** | ***Myh4*** | ***Phgdh*** |
| --- | --- | --- | --- | --- | --- |
| **Time x Treatment interaction** | | *P*=0.02 | *P*=0.008 | *P*<0.001 | *P*<0.001 |
| **Time** | **Treatment** | ***Myog*** | ***Myh7*** | ***Myh4*** | ***Phgdh*** |
| DAY 1 | Control | 1.169 | 0.184 | 0.010 | 0.096 |
| DAY 1 | 0.25 mM | 1.232 ns | 0.133 ns | 0.011 ns | 0.092 ns |
| DAY 1 | 0.5 mM | 1.123 ns | 0.105 ns | 0.011 ns | 0.092 ns |
| DAY 1 | 1 mM | 1.376 ns | 0.115 ns | 0.012 ns | 0.088 ns |
| DAY 2 | Control | 2.392 | 1.211 | 0.033 | 0.168 |
| DAY 2 | 0.25 mM | 1.824 ns | 0.803 ns | 0.061 ns | 0.191 ns |
| DAY 2 | 0.5 mM | 1.965 ns | 0.776 ns | 0.090 ns | 0.191 ns |
| DAY 2 | 1 mM | **1.242** ******* | **0.505** ****** | 0.072 ns | 0.186 ns |
| DAY 3 | Control | 1.370 | 1.487 | 0.240 | 0.161 |
| DAY 3 | 0.25 mM | 1.113 ns | 1.439 ns | **0.940** ***** | **0.094 ***** |
| DAY 3 | 0.5 mM | 0.843 ns | 1.292 ns | **1.408** ******* | **0.085 ***** |
| DAY 3 | 1 mM | 0.792 ns | 1.444 ns | **1.785** ******* | **0.079 ***** |
| DAY 4 | Control | 1.86 | 2.179 | 1.474 | 0.118 |
| DAY 4 | 0.25 mM | **0.999** ****** | 2.249 ns | **2.516** ******* | 0.121 ns |
| DAY 4 | 0.5 mM | 1.332 ns | **2.724** ***** | **3.712** ******* | **0.080 **** |
| DAY 4 | 1 mM | **1.033** ****** | 2.034 ns | **3.258** ******* | **0.069 ***** |
| DAY 5 | Control | 0.821 | 1.502 | 1.565 | 0.166 |
| DAY 5 | 0.25 mM | 0.804 ns | 1.189 ns | **2.438** ****** | **0.069 ***** |
| DAY 5 | 0.5 mM | 0.619 ns | 1.052 ns | **2.758** ******* | **0.048 ***** |
| DAY 5 | 1 mM | 0.741 ns | 1.772 ns | **2.438** ******* | **0.036 ***** |

Dunnett’s multiple comparison tests were performed on mRNA expression of genes demonstrating a significant time x treatment interaction (*P*<0.05) following treatment with 0 (Control; PBS), 0.25, 0.5 or 1mM 3-MPA from day 0 of differentiation. Data is means for n=4. Significant difference compared to control at each time-point is indicated: * P<0.05, ** P<0.01, *** P<0.001.

**Supplementary Table 3. Summary of post-hoc test analysis on mRNA expression of genes following 3-MPA treatment from day 4 of differentiation.**

Dunnett’s multiple comparison tests were performed on mRNA expression of genes demonstrating a significant time x treatment interaction (*P*<0.05) following treatment with 0 (Control; PBS), 0.25, 0.5 or 1mM 3-MPA from day 4 of differentiation. Data is means for n=4. Significant difference compared to Control at each time-point is indicated: * P<0.05, ** P<0.01, *** P<0.001.

|  |  | ***Myh7*** | ***Pck2*** | ***Phgdh*** | ***Psat1*** |
| --- | --- | --- | --- | --- | --- |
| **Time x Treatment interaction** | | *P*<0.001 | *P*=0.006 | *P*<0.001 | *P*<0.001 |
| **Time** | **Treatment** | ***Myh7*** | ***Pck2*** | ***Phgdh*** | ***Psat1*** |
| DAY 5 | Control | 0.933 | 0.771 | 0.848 | 0.969 |
| DAY 5 | 0.25 mM | 0.745 ns | 0.626 ns | **0.490** ******* | **0.655** ******* |
| DAY 5 | 0.5 mM | 0.886 ns | **0.520** ***** | **0.632** ***** | **0.467** ******* |
| DAY 5 | 1 mM | **0.638** ****** | **0.361** ******* | **0.552** ******* | **0.382** ******* |
| DAY 6 | Control | 1.311 | 0.649 | 0.895 | 0.851 |
| DAY 6 | 0.25 mM | **0.794** ******* | 0.711 ns | **0.694** ***** | 0.740 ns |
| DAY 6 | 0.5 mM | **0.473** ******* | 0.613 ns | **0.352** ******* | **0.450** ******* |
| DAY 6 | 1 mM | **0.261** ******* | 0.542 ns | **0.315** ******* | **0.387** ******* |
| DAY 7 | Control | 1.018 | 0.855 | 0.904 | 0.729 |
| DAY 7 | 0.25 mM | **0.578** ******* | **0.551** ****** | **0.412** ******* | **0.420** ******* |
| DAY 7 | 0.5 mM | **0.279** ******* | **0.480** ******* | **0.263** ******* | **0.292** ******* |
| DAY 7 | 1 mM | **0.126** ******* | **0.355** ******* | **0.228** ******* | **0.255** ******* |
| DAY 8 | Control | 1.112 | 0.596 | 0.687 | 0.471 |
| DAY 8 | 0.25 mM | **0.636** ******* | 0.733 ns | 0.570 ns | 0.558 ns |
| DAY 8 | 0.5 mM | **0.257** ******* | 0.603 ns | **0.363** ******* | 0.369 ns |
| DAY 8 | 1 mM | **0.130** ******* | 0.518 ns | **0.330** ******* | 0.332 ns |

**Supplementary Table 4. Comparison of Wildtype (WT) and Transgenic (Tg) mouse muscle tissue on PEPCK enzyme activity assay**

Protein was extracted from Wildtype (WT) or Transgenic (Tg) mouse muscle (*Tibialis Anterior, Extensor Digitorium Longus, Gastrocnemius, Quadricep*), then loaded at 20 and 40µg per well for the PEPCK activity assay (coupled to malate dehydrogenase). Representative graphs show raw data taken from Microplate Manager Software demonstrating the total time window (1,200 s) and a selected time window (0 – 300 s) with a fitted trend line. Mean velocity and initial absorbance were calculated from the slope and intercept respectively. Means, standard error of mean (SEM) and coefficient of variance (CV; %) are presented.

|  | Protein quantity (µg) | **Mean velocity** (mOD/min) | SEM | CV (%) | N | **Representative graph** | **Mean absorbance** (mOD) | SEM | CV (%) | N |
| --- | --- | --- | --- | --- | --- | --- | --- | --- | --- | --- |
| WT | 20 | 1.37 | 0.09 | 11.18 | 3 | **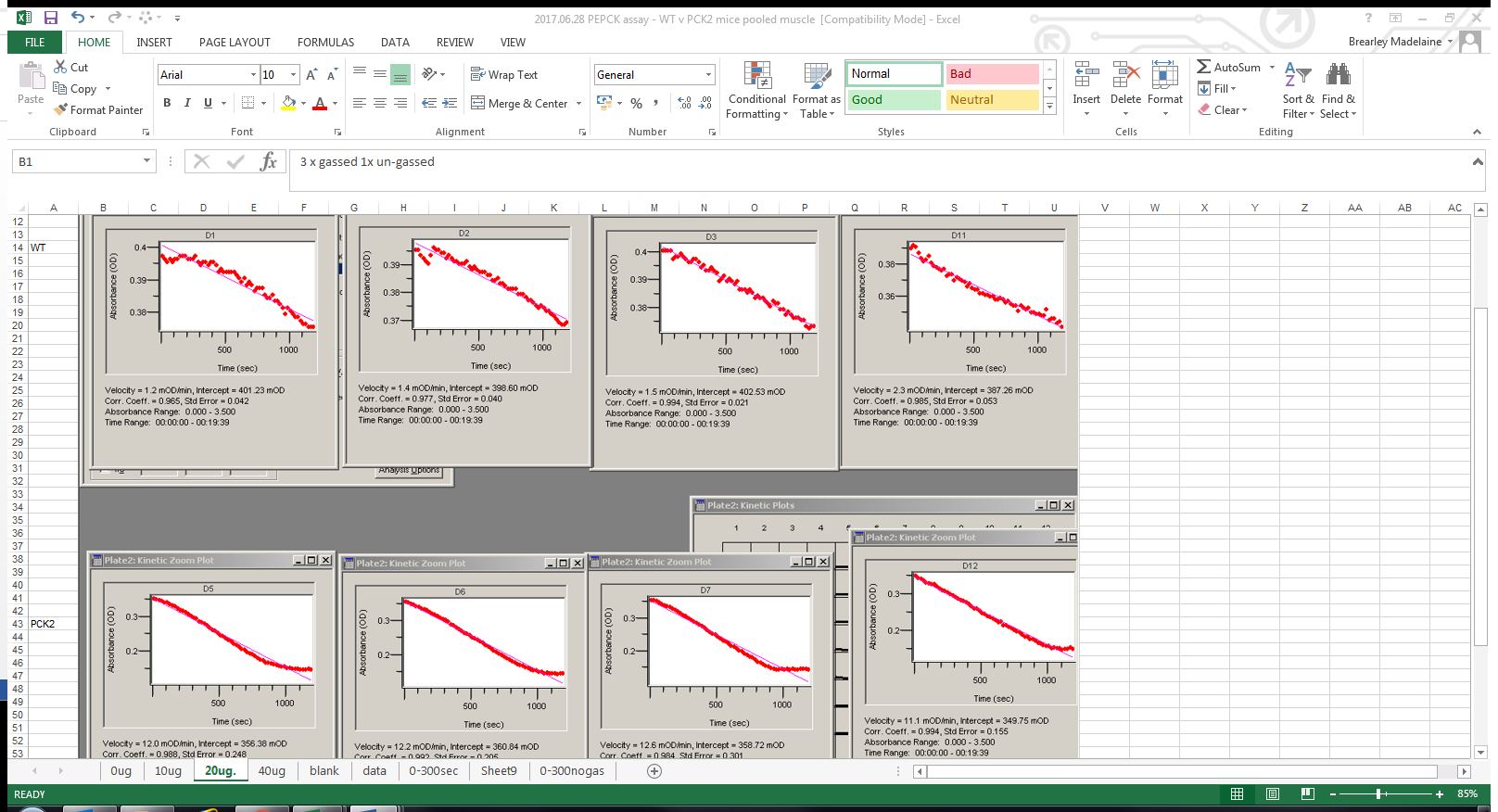** | 400.8 | 1.56 | 0.50 | 3 |
| Tg | 20 | 12.27 | 0.18 | 2.49 | 3 | **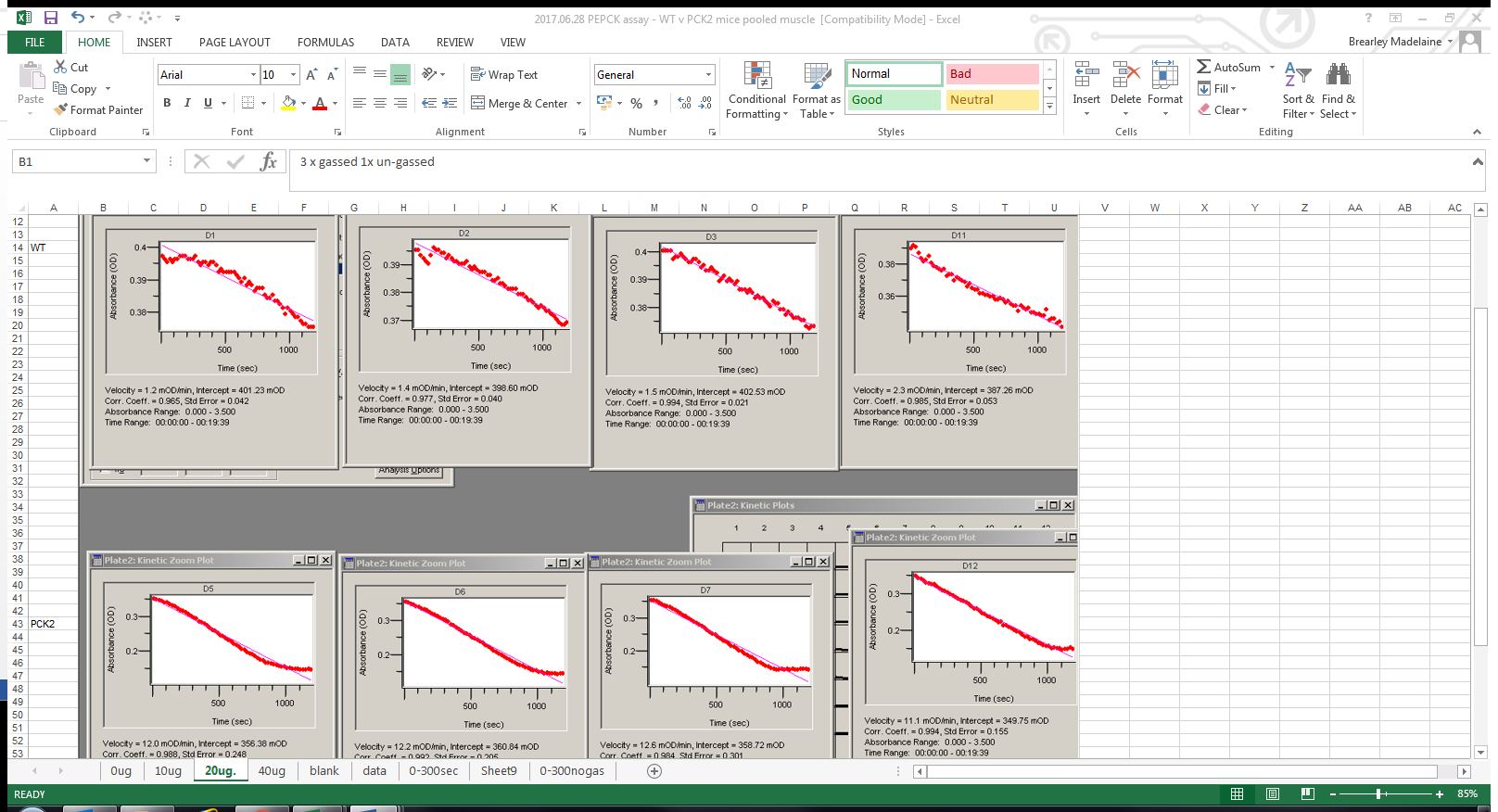** | 358.6 | 1.29 | 0.62 | 3 |
| WT | 40 | 3.97 | 0.15 | 6.34 | 3 | **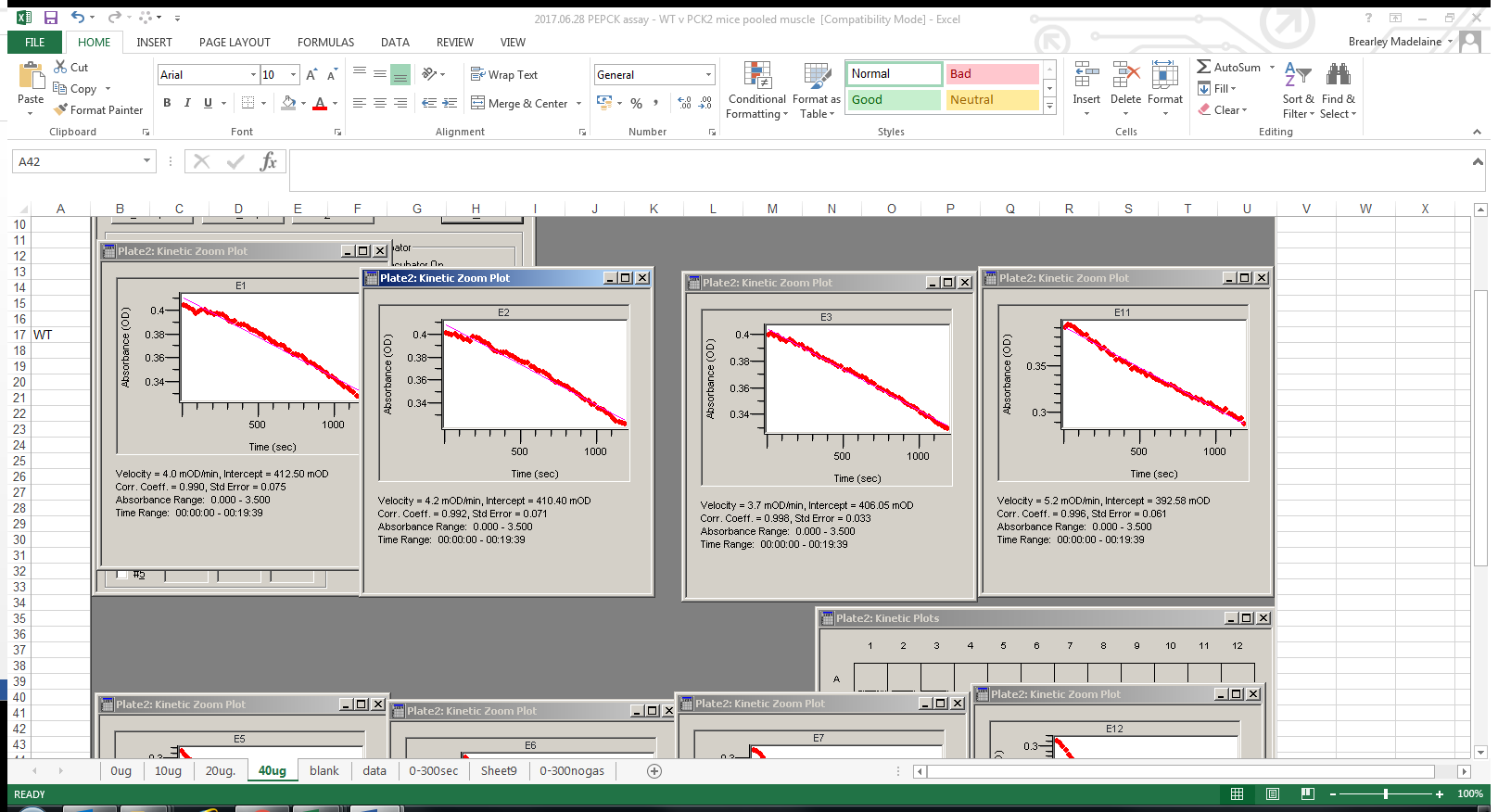** | 409.7 | 1.90 | 0.80 | 3 |
| Tg | 40 | 7.57 | 0.12 | 2.75 | 3 | 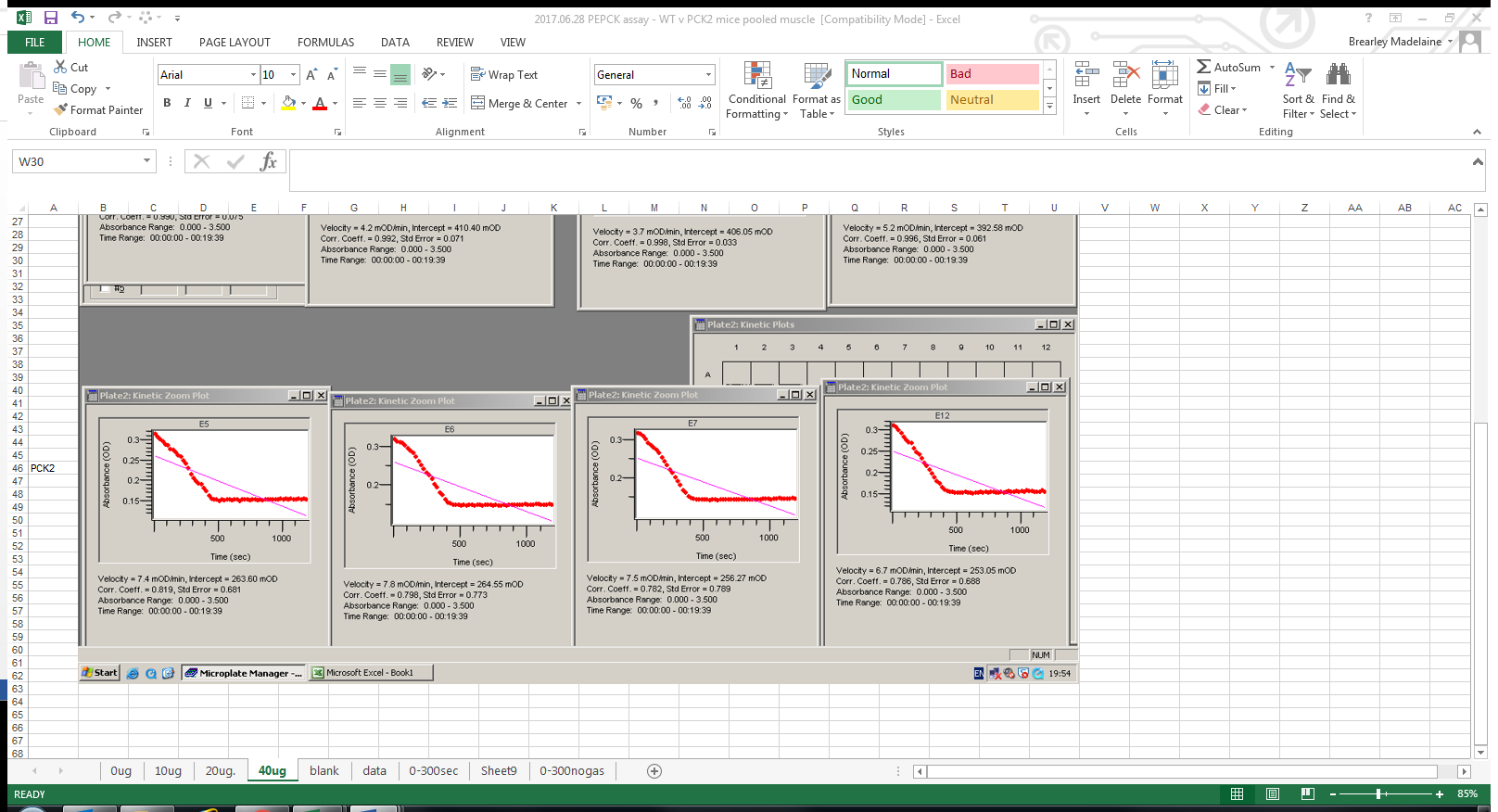 | 261.5 | 2.62 | 1.73 | 3 |
| WT | 40 | 2.27 | 0.32 | 24.30 | 3 | **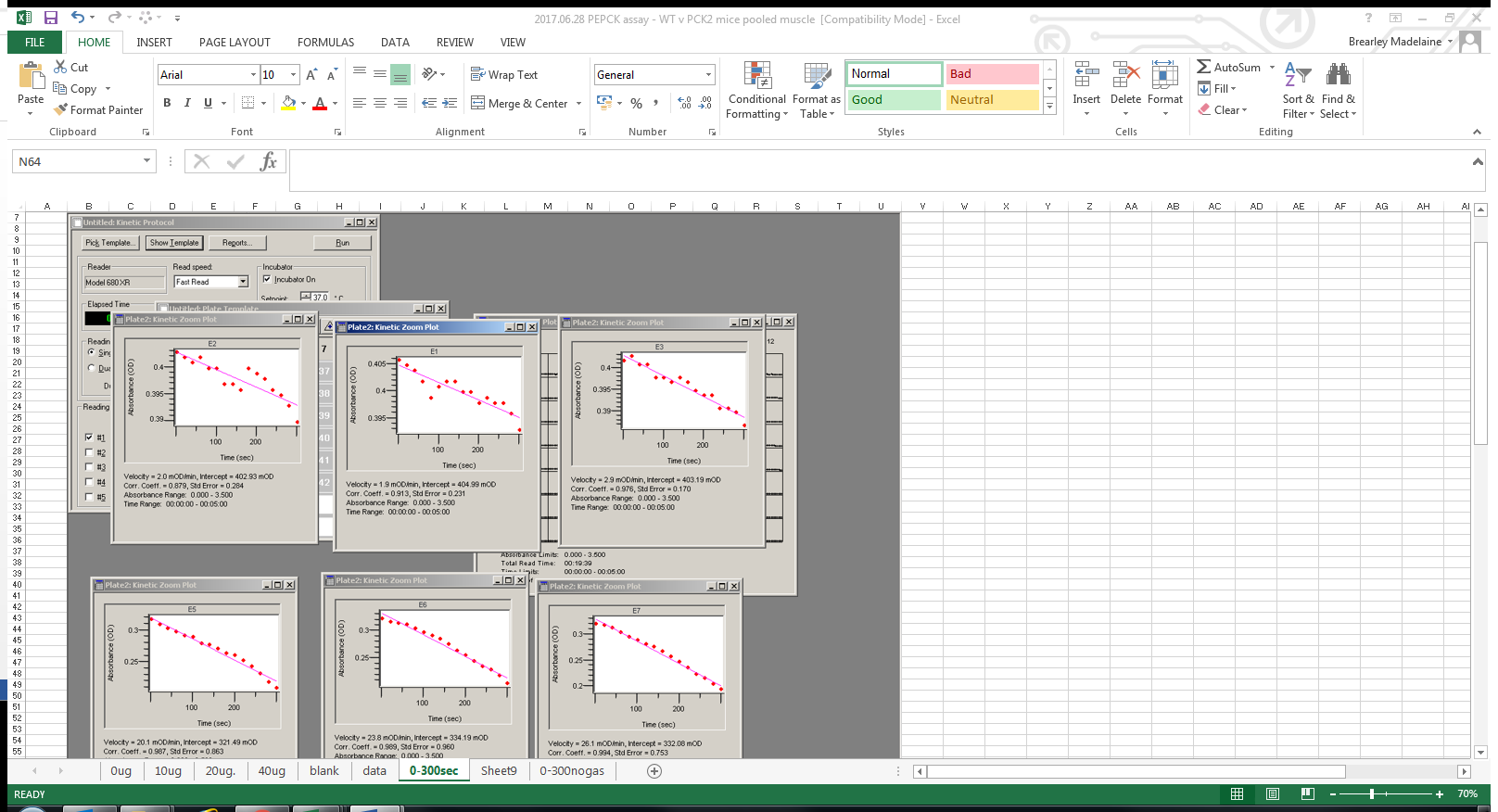** | 403.7 | 0.65 | 0.28 | 3 |
| Tg | 40 | 23.33 | 1.75 | 12.97 | 3 | **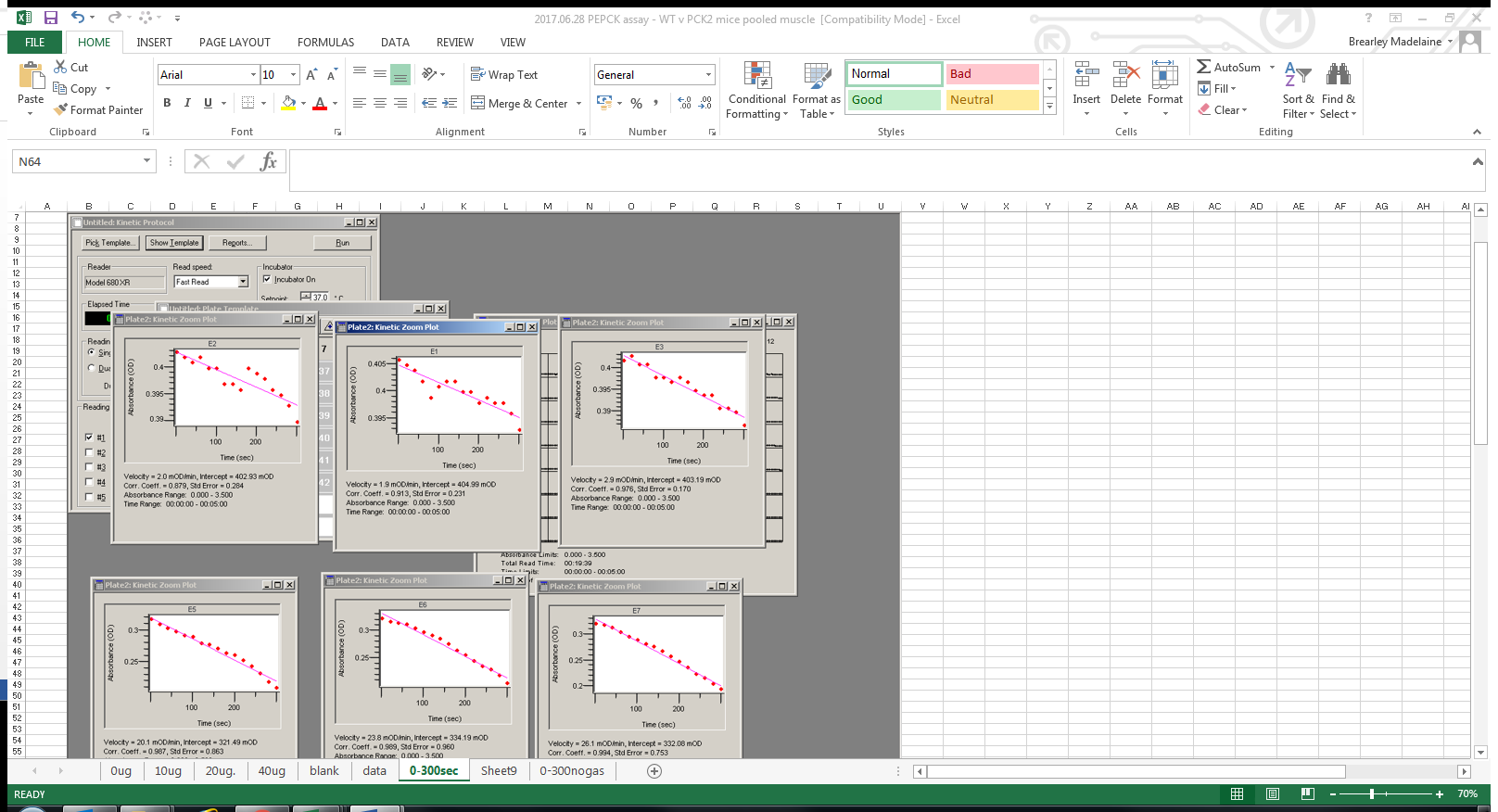** | 329.3 | 3.93 | 2.07 | 3 |

**Supplementary Table 5. Effect of 3-MPA on velocity and initial absorbance of PEPCK activity assay in the absence of different assay components**

PEPCK enzymatic activity assay was performed using a range of 3-MPA doses (0-1mM) with: (A) all assay mastermix components, except protein; (B) assay mastermix components, except protein or NADH; (C) no protein, NADH or assay mastermix components, i.e. HEPES buffer only. Mean velocity and absorbance were calculated from the slope and intercept respectively. Means, standard error of mean (SEM) and coefficient of variance (CV; %) are presented for n=2-4, as stated below. ‘No data’ indicates samples where a line, and therefore a slope to calculate velocity, could not be determined. Although the assay was performed in the absence of protein, mean velocity was increased with increasing dose of 3-MPA for conditions (A) and (B), with the exception of the highest dose (1mM) where velocity could not be calculated. In all cases (A, B and C) initial absorbance was increased with increasing dose of 3-MPA demonstrating that 3-MPA potentially absorbs at the same wavelength used in the PEPCK activity assay to quantify depletion of NADH (340nm).

|  | **(A) No protein** | | |  | **(B) No protein or NADH** | | |  | **(C) HEPES only** | | | |
| --- | --- | --- | --- | --- | --- | --- | --- | --- | --- | --- | --- | --- |
| 3-MPA (mM) | **Mean velocity** (mOD/min) | SEM | CV (%) | N | **Mean velocity** (mOD/min) | SEM | CV (%) | N | **Mean velocity** (mOD/min) | SEM | CV (%) | N |
| 0 | **0.65** | 0.06 | 19.86 | 4 | No data | - | - | - | **0.30** | 0.15 | 88.19 | 3 |
| 0.1 | **1.23** | 0.30 | 49.60 | 4 | **0.48** | 0.18 | 73.68 | 4 | **0.17** | 0.09 | 91.65 | 3 |
| 0.25 | **1.83** | 0.11 | 12.15 | 4 | **0.88** | 0.21 | 47.01 | 4 | **0.30** | 0.08 | 54.43 | 4 |
| 0.5 | **3.85** | 0.10 | 5.41 | 4 | **2.03** | 0.20 | 19.91 | 4 | 0 | 0 | 0 | 2 |
| 1 | No data | - | - | - | No data | - | - | - | No data | - | - | - |
|  |  |  |  |  |  |  |  |  |  |  |  |  |
| 3-MPA (mM) | **Mean absorbance** (mOD) | SEM | CV (%) | N | **Mean absorbance** (mOD) | SEM | CV (%) | N | **Mean absorbance** (mOD) | SEM | CV (%) | N |
| 0 | 442.35 | 1.13 | 0.51 | 4 | 133.75 | 1.65 | 2.47 | 4 | 125.35 | 0.88 | 1.40 | 4 |
| 0.1 | 498.22 | 5.09 | 2.05 | 4 | 190.35 | 0.63 | 0.66 | 4 | 189.23 | 2.24 | 2.36 | 4 |
| 0.25 | 598.94 | 3.38 | 1.13 | 4 | 298.40 | 0.52 | 0.35 | 4 | 276.75 | 1.60 | 1.16 | 4 |
| 0.5 | 751.41 | 2.16 | 0.58 | 4 | 455.89 | 0.87 | 0.38 | 4 | 437.24 | 2.21 | 1.01 | 4 |
| 1 | 1050.00 | 4.08 | 0.78 | 4 | 781.25 | 3.15 | 0.81 | 4 | 678.24 | 1.21 | 0.36 | 4 |

**Supplementary Table 6. Representative graphs of PEPCK activity output with increasing doses of 3-MPA**

PEPCK enzymatic activity assay was performed using a range of 3-MPA doses (0-1mM) with: (A) all assay mastermix components, except protein; (B) assay mastermix components, except protein or NADH; (C) no protein, NADH or assay mastermix components, i.e. HEPES buffer only. Representative graphs of raw data outputs taken from the Microplate Manager Software to illustrate the effect of increasing doses of 3-MPA on initial absorbance and velocity of PEPCK activity assay with no protein present. The positive slope observed with the highest dose (1mM) demonstrates that a product might be performed from the degradation of 3-MPA, which is also absorbed at the same wavelength used in the PEPCK activity assay to quantify degradation of NADH (340nm).

| 3-MPA (mM): | **0** | **0.1** | **0.25** | **0.5** | **1** |
| --- | --- | --- | --- | --- | --- |
| **(A)**  **- Protein**  **+ NADH**  **+ Mastermix** | **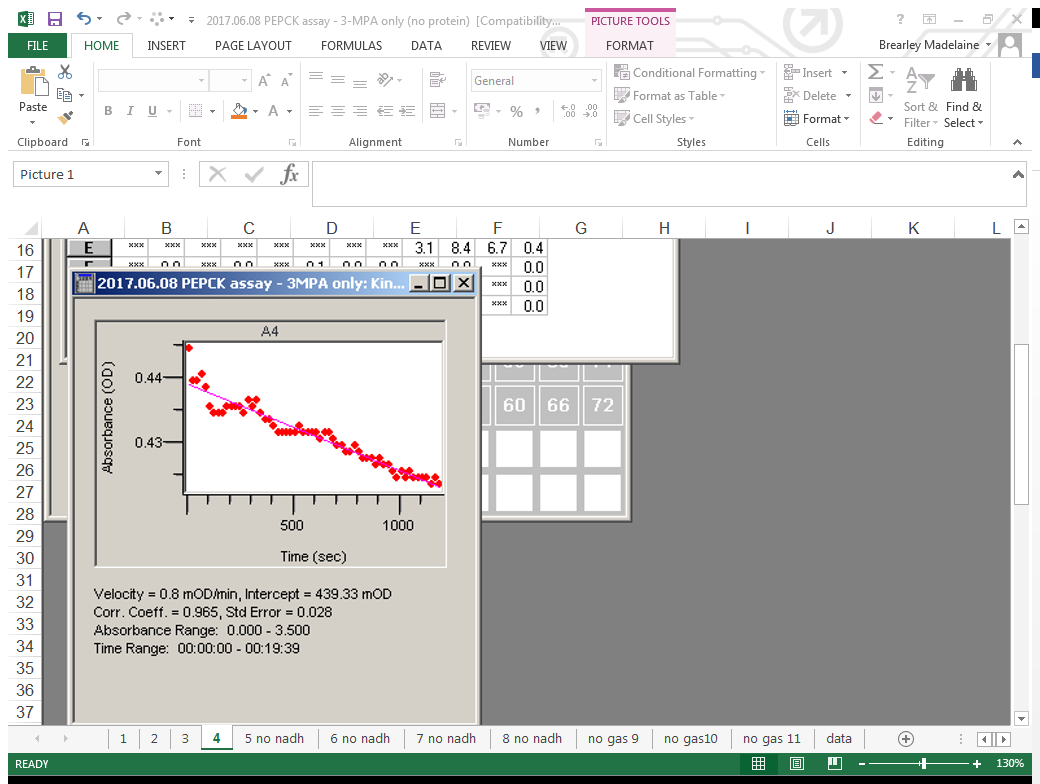** | **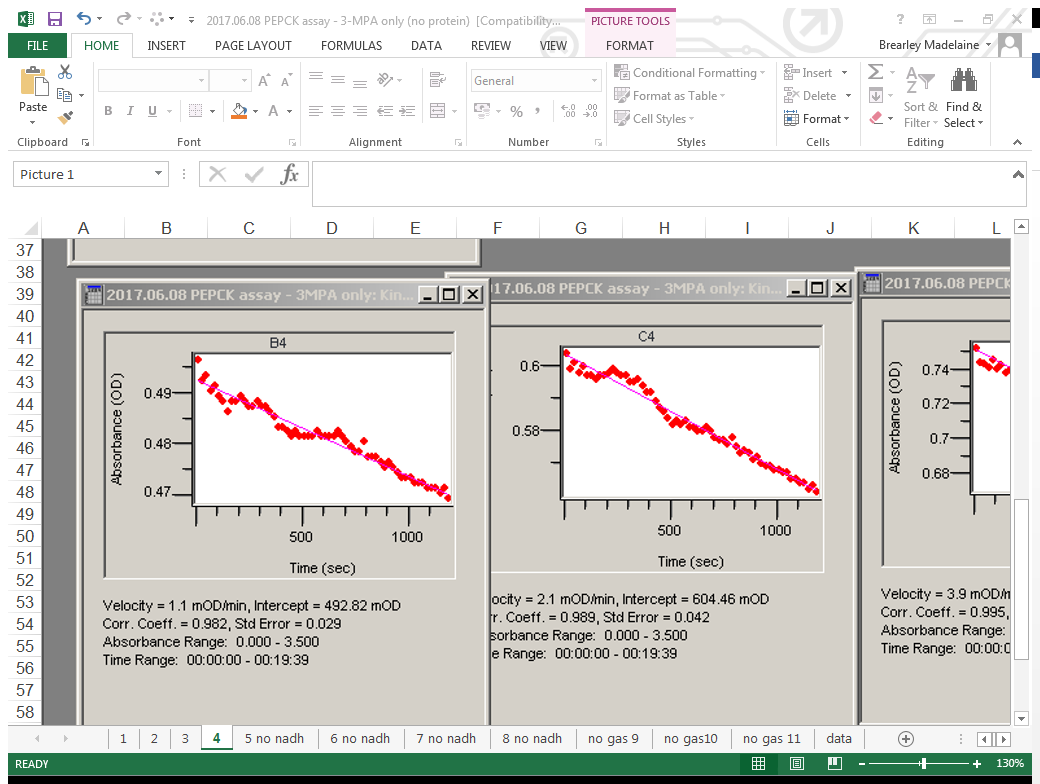** | **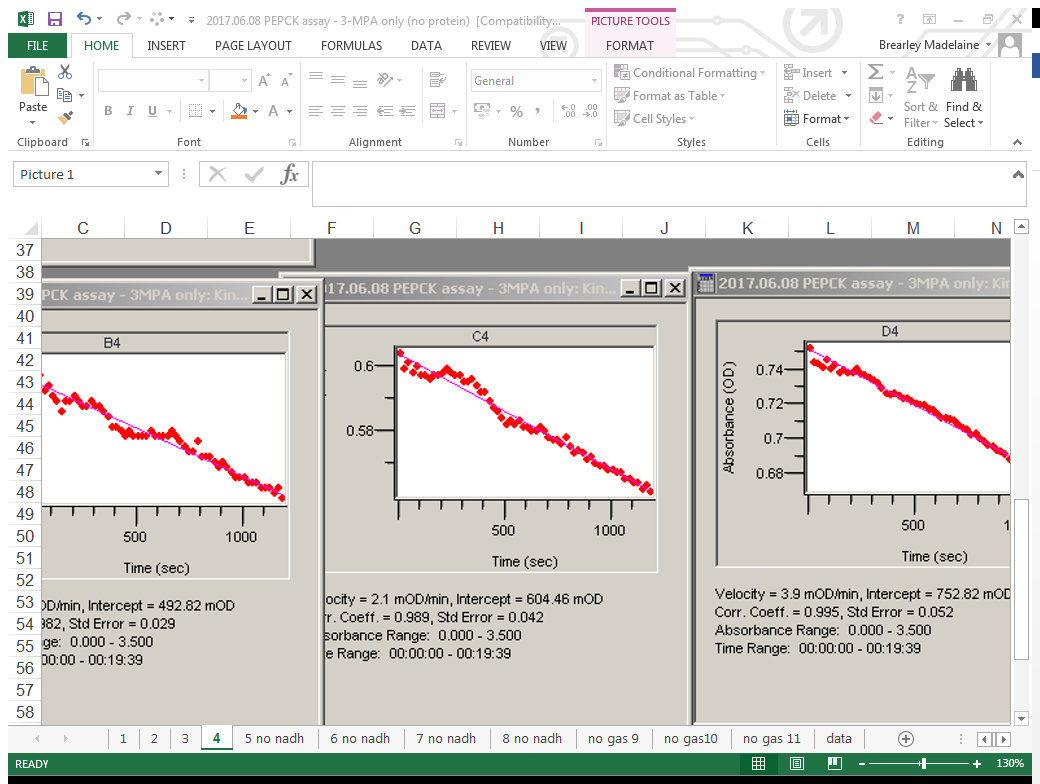** | **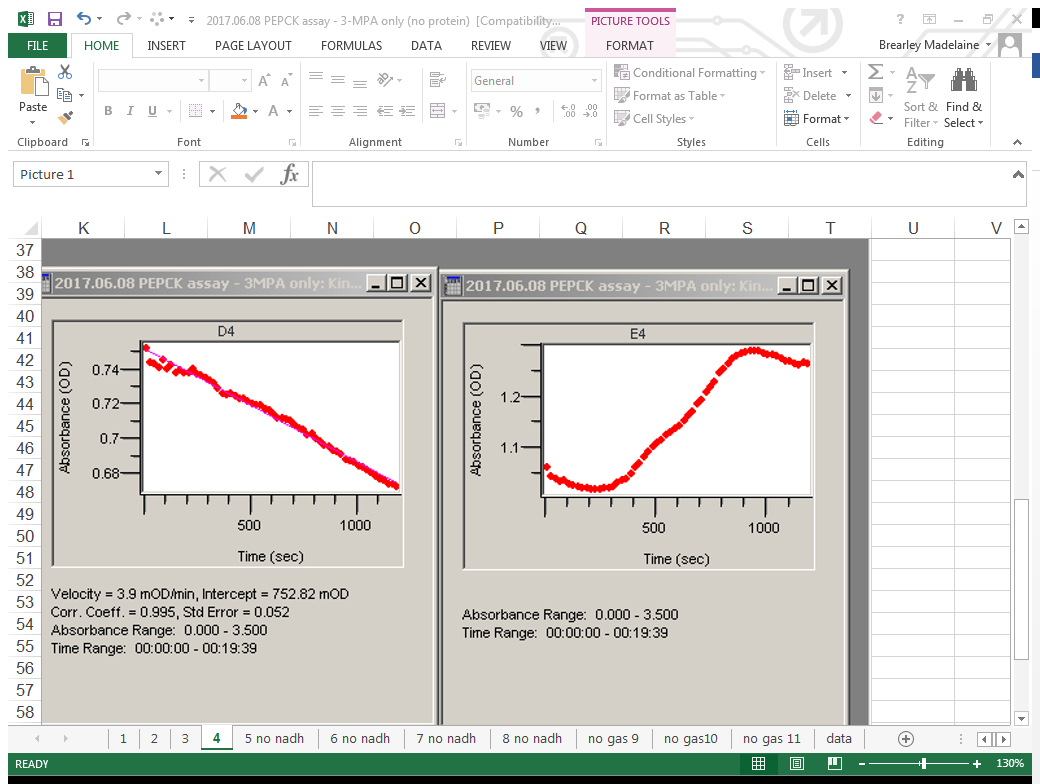** | **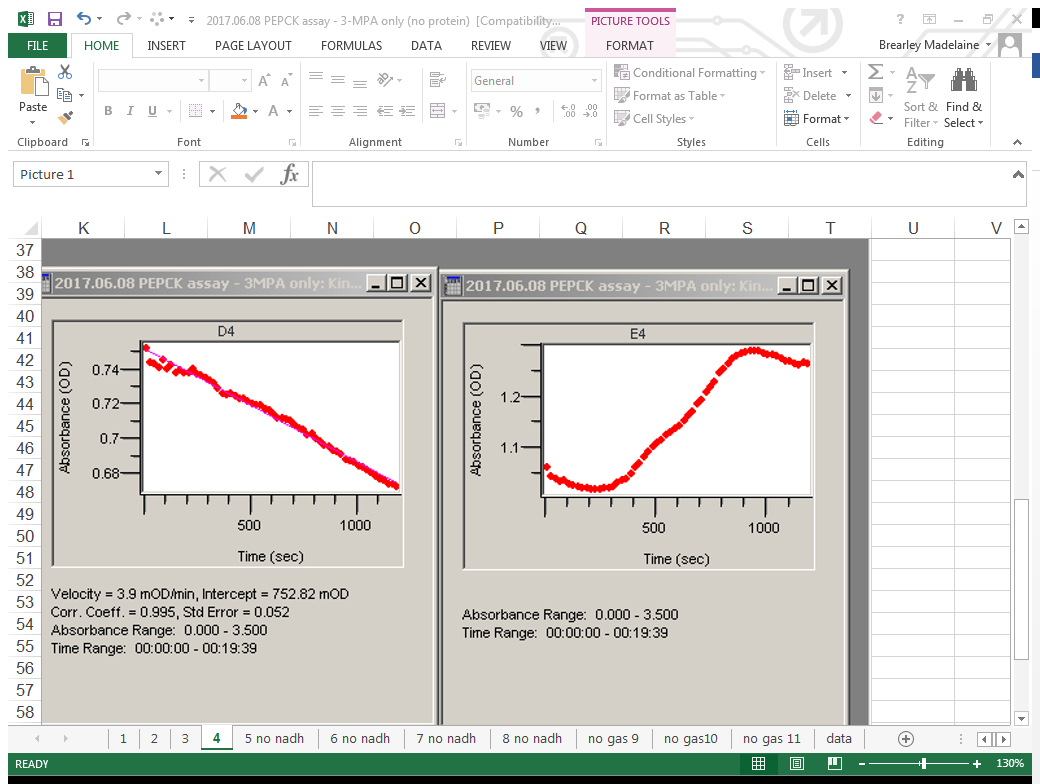** |
| **(B)**  **- Protein**  **- NADH**  **+ Mastermix** | **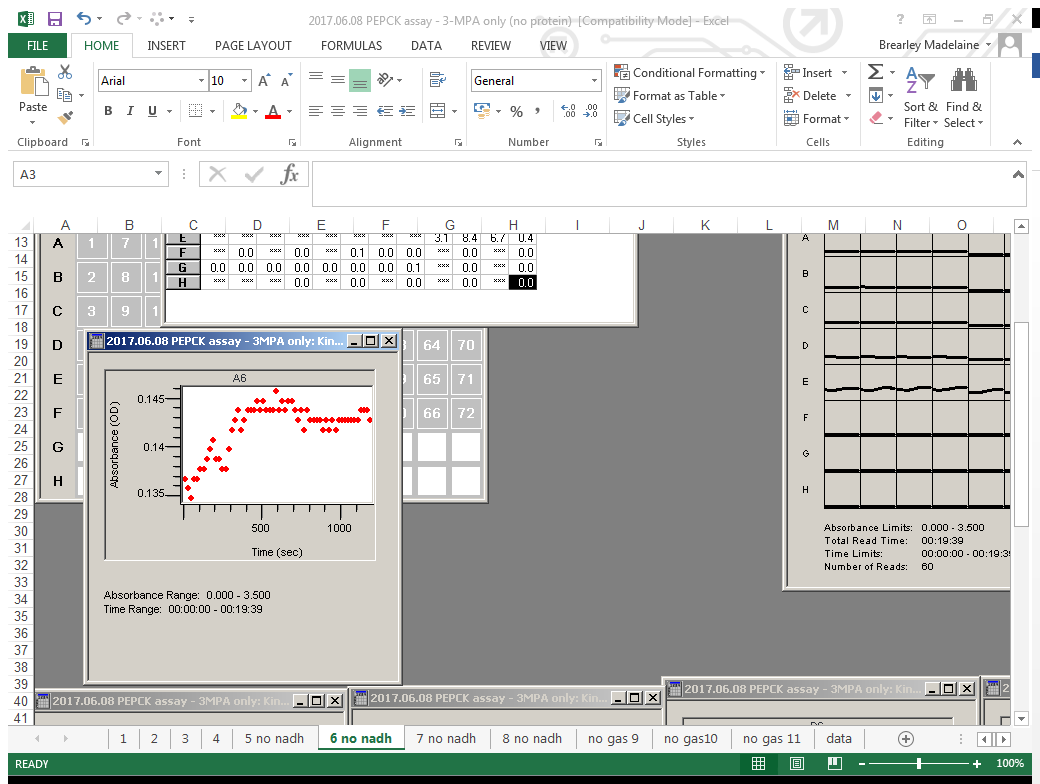** | **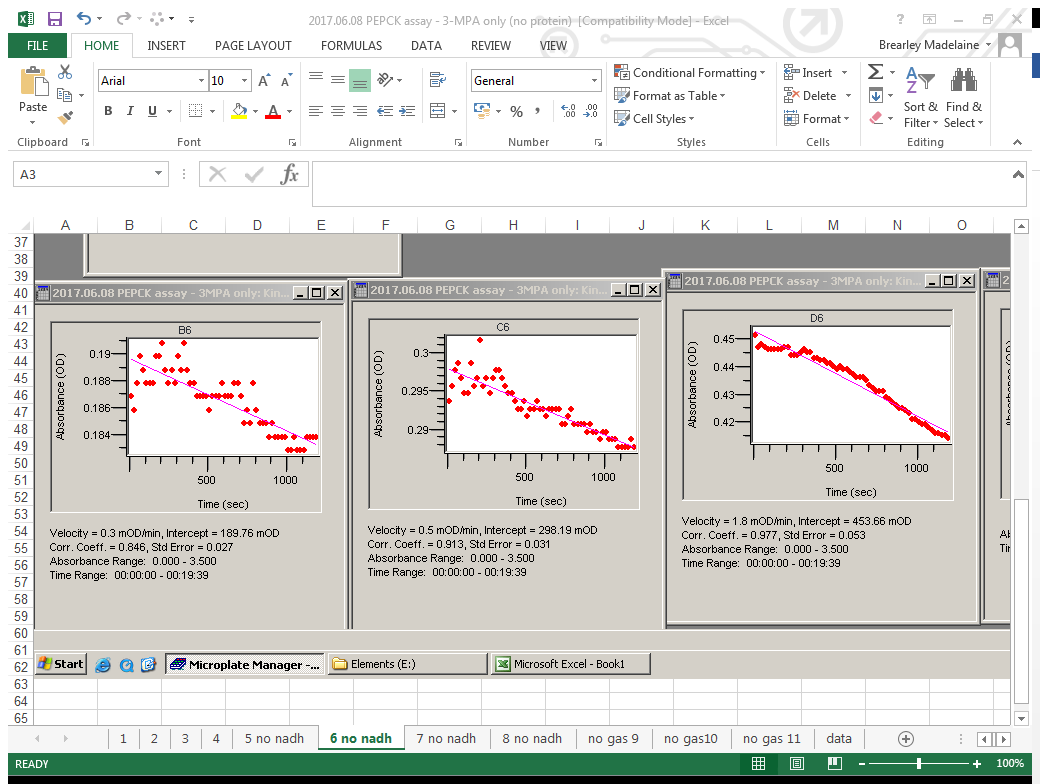** | **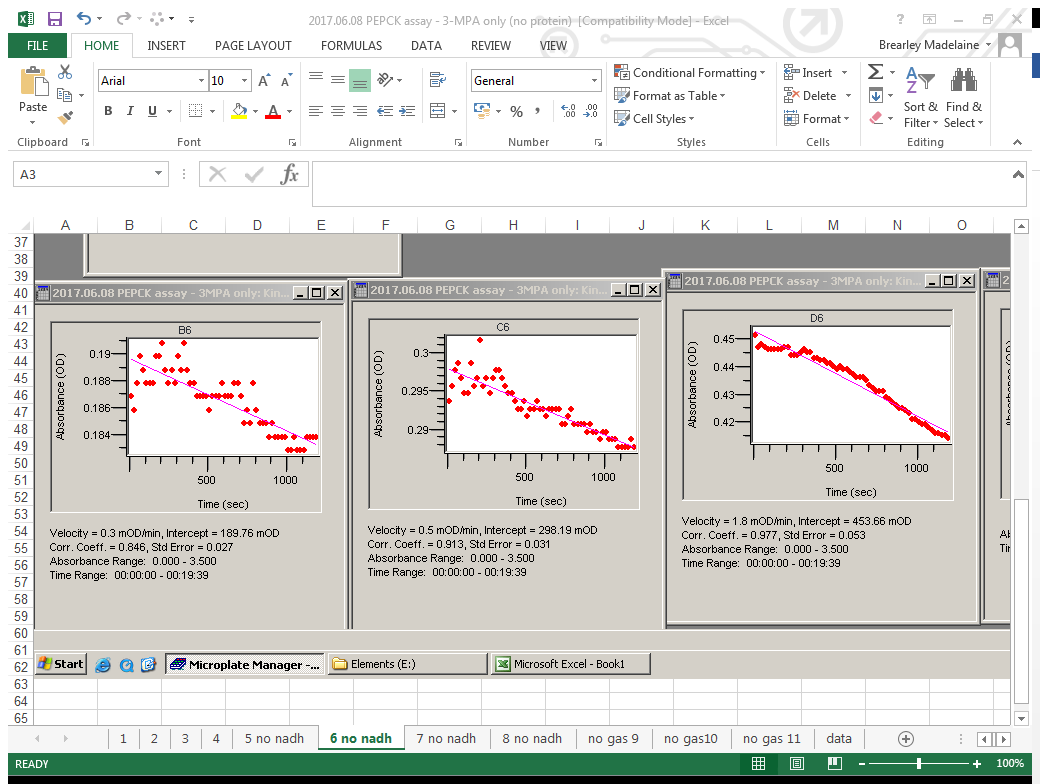** | **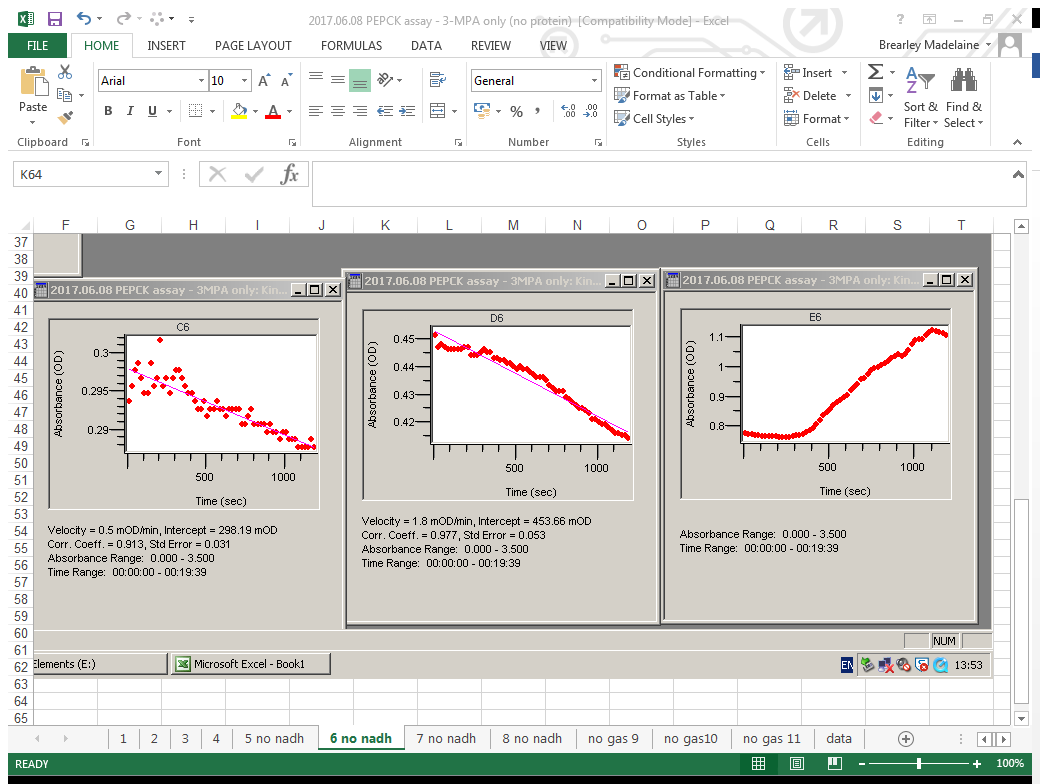** | **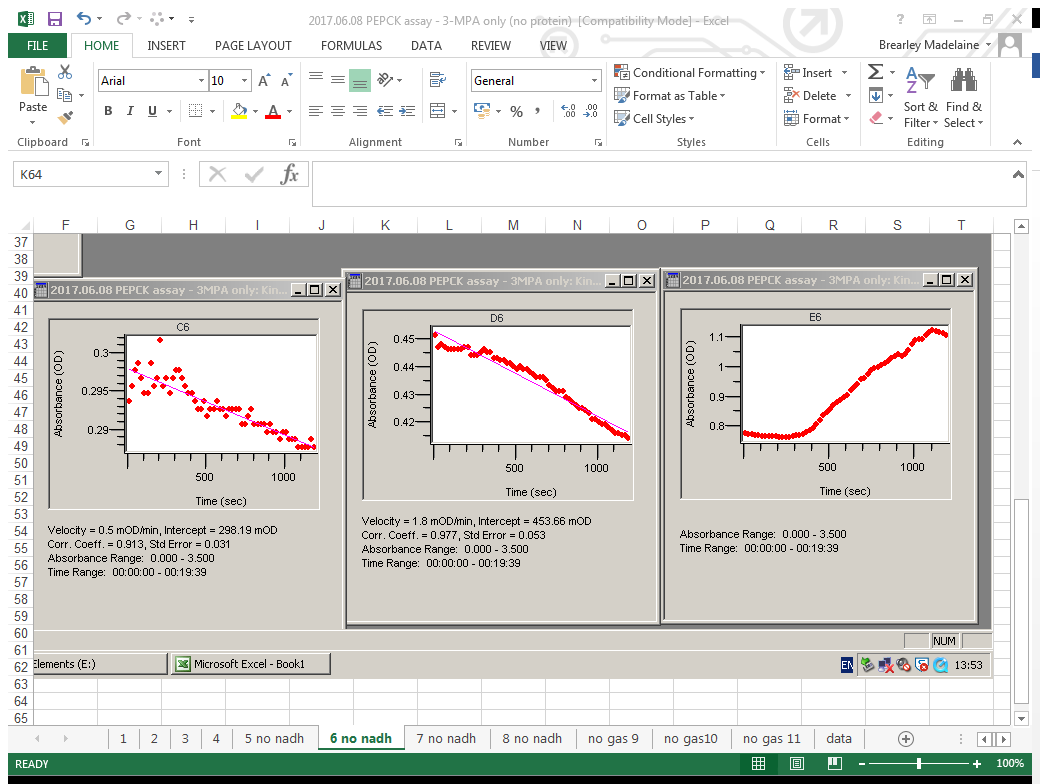** |
| **(C)**  **- Protein**  **- NADH**  **- Mastermix** | 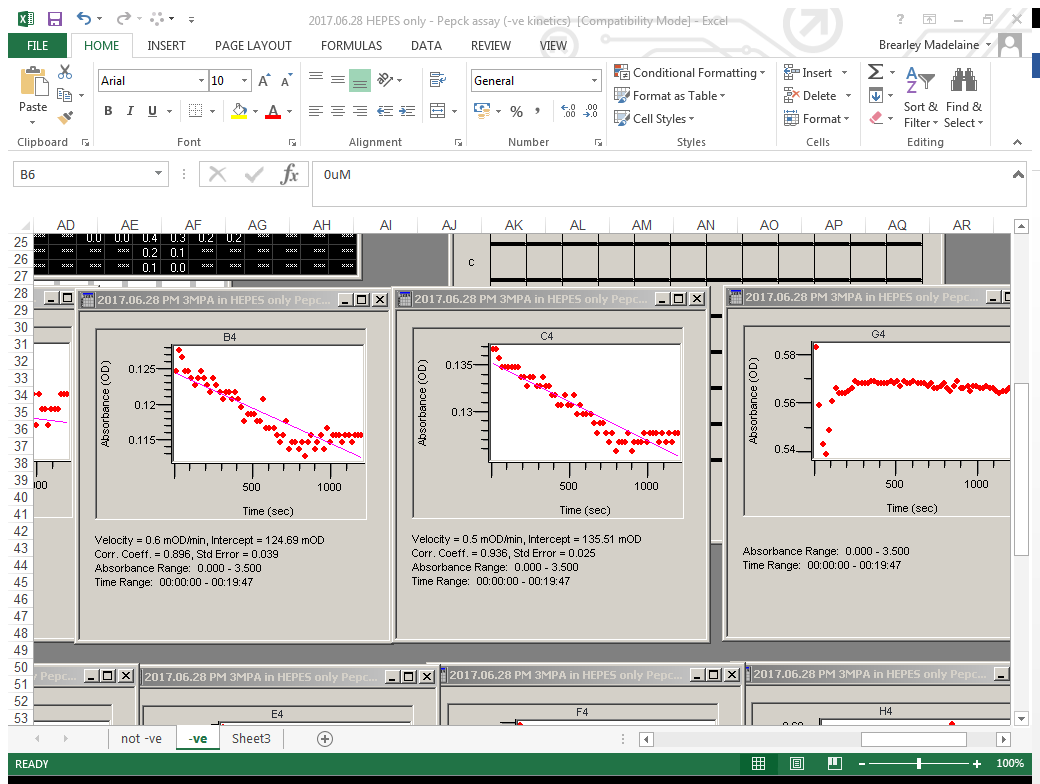 | 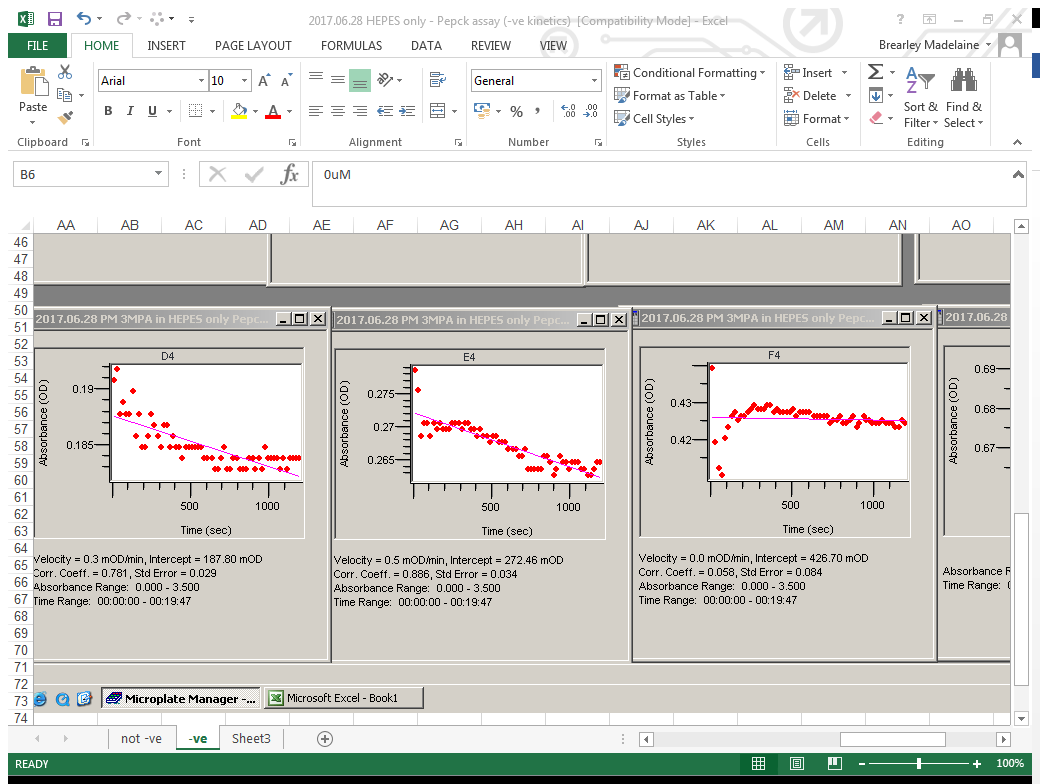 | 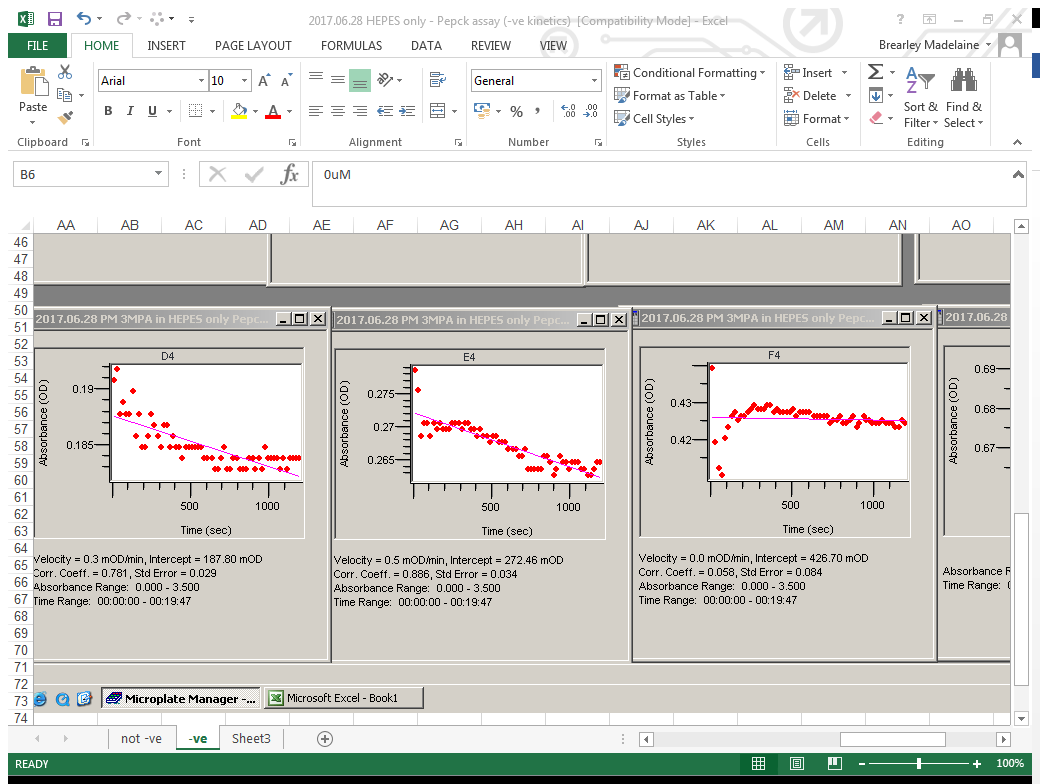 | 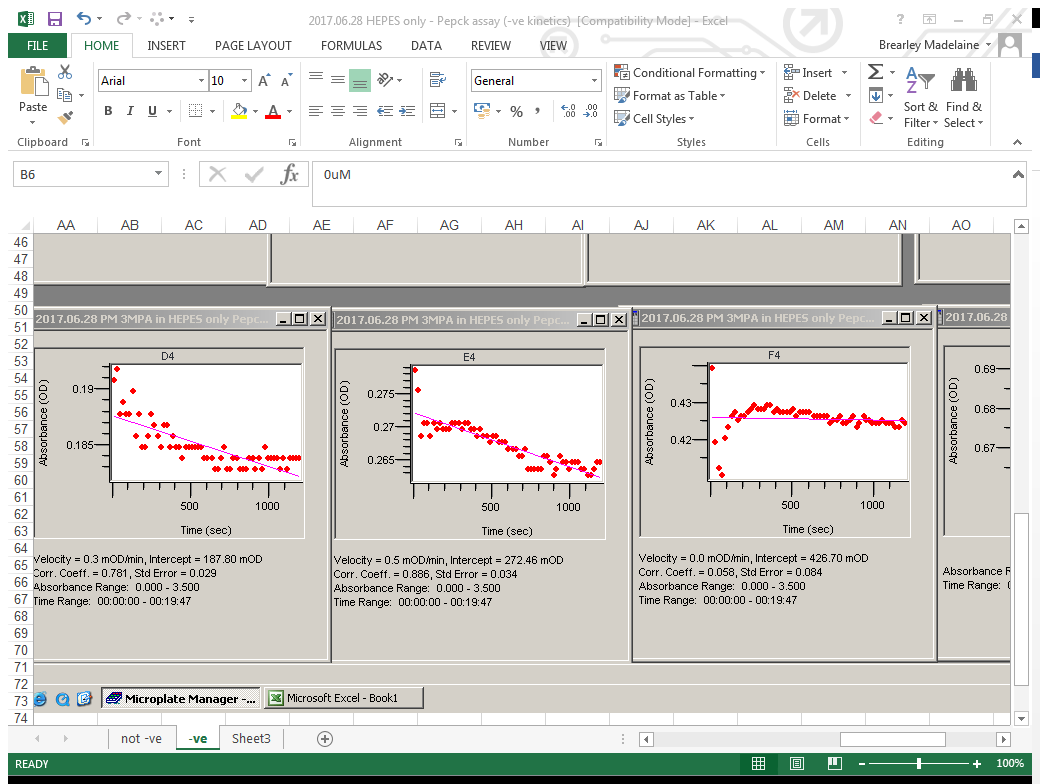 | 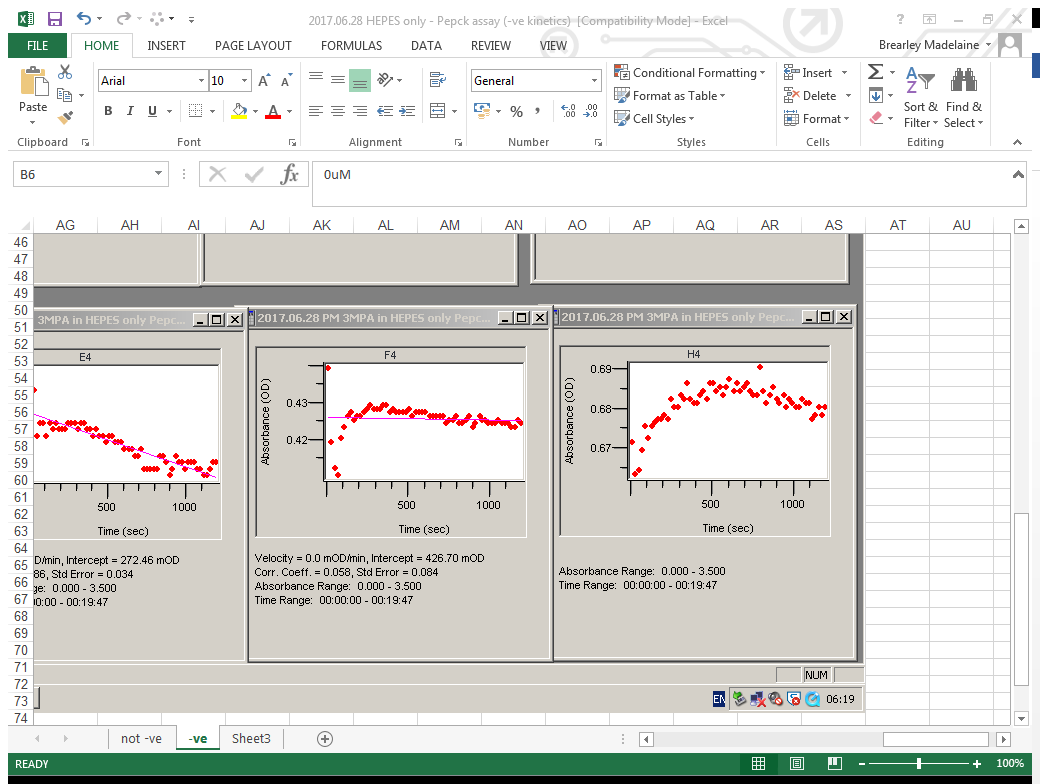 |

**Supplementary Table 7. Forward and reverse murine primer sequences for QPCR**

| **Gene** | **Forward primer (5’ 🡪 3’)** | **Reverse primer (5’ 🡪 3’)** |
| --- | --- | --- |
| *Myog*^1^ | CCCATGGTGCCCAGTGAA | GCAGATTGTGGGCGTCTGTA |
| *Myh7*^2^ | CTCAAGCTGCTCAGCAATCTATTT | GGAGCGCAAGTTTGTCATAAGT |
| *Myh4*^3^ | CAATCAGGAACCTTCGGAACAC | GTCCTGGCCTCTGAGAGCAT |
| *Pck1* | CCCAAGGCAACTTAAGGGCTAT | CTGAGGTGCCAGGAGCAACT |
| *Pck2*^4,5^ | GCAGAGCACATGCTGATTTTG | GGAAAGCAGCTGCCACGTA |
| *Phgdh*^4,5^ | CGTGAACTTGGTGAACGCTAAG | GTGGGAGGTGGTGACATTGAG |
| *Psat1*^4,5^ | CGTGCTTCAGCATCTACGTCAT | GCCCCGCCGTTGTTCT |

**References**

1. Brown, D. M., Parr, T. & Brameld, J. M. Myosin heavy chain mRNA isoforms are expressed in two distinct cohorts during C2C12 myogenesis. *J. Muscle Res. Cell Motil.* **32**, 383–90 (2012).

2. Zhou, Y., Liu, D. & Kaminski, H. J. Myosin heavy chain expression in mouse extraocular muscle: more complex than expected. *Invest. Ophthalmol. Vis. Sci.* **51**, 6355–63 (2010).

3. da Costa, N. *et al.* Calcineurin differentially regulates fast myosin heavy chain genes in oxidative muscle fibre type conversion. *Cell Tissue Res.* **329**, 515–27 (2007).

4. Brown, D. M. *et al.* Effect of sodium 4-phenylbutyrate on Clenbuterol-mediated muscle growth. *PLoS One* **13**, e0201481 (2018).

5. Brearley, M. C. *et al.* Changes in expression of serine biosynthesis and integrated stress response genes during myogenic differentiation of C2C12 cells. *Biochem. Biophys. Reports* **20**, 100694 (2019).
